# Cultivar-Dependent Metabolite Signatures and Effects on Lipid Metabolism of Teas Processed from Three Varieties of *Toona sinensis*

**DOI:** 10.3390/metabo16100760

**Published:** 2026-10-09

**Authors:** Guohuo Wu, Yan Tang, Shuolei Xu, Zhaofeng Du, Ruojue Xu, Boyu Du, Guangshun Lu, Xiaoyun Wu, Juanjuan Sui

**Affiliations:** Engineering Technology Research Center of Anti-Aging Chinese Herbal Medicine of Anhui Province, School of Biology and Food Engineering, Fuyang Normal University, Fuyang 236037, China

**Keywords:** *Toona sinensis*, tea, cultivar, metabolomics, lipid metabolism, flavonoids

## Abstract

**Background:** The edible and medicinal plant *Toona sinensis* holds promise for functional food development, yet how cultivar variation affects the metabolite profiles and lipid-modulating effects of its processed leaf teas remains poorly understood. **Methods:** We systematically prepared and compared teas from three dominant cultivars—Green Toon (GT), Red Toon (RT), and Black Toon (BT)—using a standardized black tea processing procedure. Untargeted metabolomics combined with chemometric analysis was used to characterize cultivar-dependent metabolite signatures. Quantitative analysis was performed for total flavonoids, total polyphenols, and free amino acids. The lipid-modulating effects were evaluated in high-fat-diet-fed mice after 12-week oral administration of RT or BT (equivalent to 2.67 mg/g body weight per day, corresponding to ~14 g tea/day for a 60 kg adult), with GT as the comparison group. **Results:** Untargeted metabolomics with chemometric analysis revealed distinct cultivar-dependent metabolite signatures. RT and BT contained significantly higher levels of flavonoids and phenolic acids than GT. Notably, BT was putatively rich in flavonoid sulfates and chlorogenic acid derivatives, whereas RT putatively accumulated acylated flavonoid glycosides and theogallin. Quantitative analysis confirmed that RT and BT had higher contents of total flavonoids, total polyphenols, and free amino acids than GT. In high-fat-diet-fed mice, 12-week oral administration of RT or BT markedly attenuated body weight gain, hepatic steatosis, and dyslipidemia (improving triglycerides, total cholesterol, and LDL cholesterol), while GT showed only mild beneficial effects. Correlation analysis further demonstrated that polyphenols and flavonoids, particularly quercetin derivatives and specific phenolic acids, were significantly correlated with improvements in lipid metabolism parameters. **Conclusions:** These results highlight that cultivar selection is strongly associated with the chemical composition and lipid-regulatory effects of *T. sinensis* leaf teas, and suggest RT and BT as superior cultivars for the development of functional teas targeting metabolic health; these findings provide actionable data for evidence-based cultivar selection in the food industry.

## 1. Introduction

*Toona sinensis* (A. Juss.) Roem is a perennial deciduous tree widely distributed across Asia, with abundant germplasm resources particularly in China. As a multi-functional economic species, it has been extensively utilized for edible, medicinal, and timber purposes [1]. Although *T. sinensis* is native to Asia, it has been introduced to other continents as an ornamental tree, and its shoots are now exported to countries in Europe, North America, and Australia. Its tender buds and leaves, serving as traditional forest vegetables for more than one thousand years, are rich in essential amino acids, vitamins, and polyunsaturated fatty acids, thus exhibiting outstanding nutritional value [2]. Modern studies have validated that *T. sinensis* extracts possess prominent antioxidant, anti-inflammatory, and hepatoprotective activities, as well as favorable regulatory effects on glucose and lipid metabolism [3]. Notably, its antioxidant capacity ranks higher than that of blueberries and most other common fruits and vegetables [4]. Flavonoids, especially flavonols, are recognized as the core bioactive constituents supporting its nutritional and health-promoting functions. To date, 19 flavonoids have been identified from *T. sinensis* buds, among which quercetin and its glycosides are the most representative components [3].

However, fresh tender shoots are highly perishable and available only in spring, which severely restricts their year-round utilization and commercial scalability. In response, processing *T. sinensis* leaves into herbal tea has emerged as a practical strategy for preservation, deep processing, and high-value utilization. Our research group previously introduced black tea processing technology into *T. sinensis* leaves and successfully developed a novel *T. sinensis* leaf tea (TLT) [5]. Preliminary results showed that, compared with direct drying of fresh leaves, TLT displayed significantly increased contents of flavonoids, flavonoid glycosides, and free amino acids, together with improved flavor and sensory palatability. In recent years, such tea products have attracted growing attention as functional beverages. Notably, although *T. sinensis* leaves are processed into tea, they differ fundamentally from conventional tea (*Camellia sinensis*) in botanical origin, characteristic bioactive compounds, caffeine content, and flavor profile (Table 1). Unlike the catechin- and caffeine-rich conventional tea, *T. sinensis* leaf tea is characterized by flavonol glycosides and terpenoids, with low or negligible caffeine and a unique pungent aroma. A large number of studies have confirmed that plant-derived foods are remarkably cultivar-dependent in their metabolite profiles and bioactivities [6,7,8]. These compositional differences from conventional tea further imply that cultivar variation may profoundly affect the metabolite signatures and health-promoting properties of *T. sinensis* leaf tea.

In China, the nutritious buds with distinct flavors are the mainstay of the *T. sinensis* industry, with over 1.3 × 10^5^ hectares cultivated specifically for bud harvesting. This substantial cultivation scale underscores the economic importance of *T. sinensis* and the growing interest in its processing and value-added utilization [13]. Long-term natural evolution and artificial domestication have endowed *T. sinensis* with rich germplasm resources [14], which are phenotypically distinguished by bud colors ranging from green to reddish-brown and deep purple-red [15]. Among them, ‘Red Toon’ (buds in deep red), ‘Black Toon’ (buds ranging from deep purple to black), and ‘Green Toon’ (green buds with faint red tips) have become the dominant commercial cultivars due to their stable phenotypes, distinctive flavors, and strong adaptability.

Nevertheless, existing comparative studies have focused almost exclusively on the nutritional composition of raw tender shoots. Furthermore, although our group has recently investigated the effects of processing methods (direct drying vs. black tea processing) and harvest maturity (tender leaves vs. mature leaves) on *T. sinensis* leaf tea [5,16]—both studies using a single cultivar (‘Black Toon’)—the metabolite profiles and lipid-modulating effects of teas processed from different *T. sinensis* cultivars remain completely unexplored. In particular, no study has systematically linked cultivar-dependent chemical signatures to in vivo metabolic outcomes. This research gap directly restricts the screening of elite cultivars for tea processing and the development of high value-added functional products.

Meanwhile, the growing global prevalence of diet-induced lipid metabolism disorders has boosted the demand for natural, safe, and food-derived strategies to maintain metabolic health [17]. Plant-origin beverages, especially teas, have attracted extensive attention owing to their rich polyphenol profiles and long history of safe consumption [18]. Although pharmacological interventions have made certain progress in managing metabolic disorders, long-term application may lead to potential side effects, which underscores the urgency of developing natural and sustainable dietary alternatives [19]. Accordingly, exploiting herbal teas as daily dietary supplements to regulate lipid metabolism has become a research frontier in food science and nutrition.

Given that anthocyanins and flavonol glycosides are often more abundant in red or purple plant tissues, we hypothesized that ‘Red Toon’ and ‘Black Toon’ teas would exhibit superior antioxidant and lipid-lowering activities compared with ‘Green Toon’ tea. To test this hypothesis, the present study selected three representative cultivars—‘Red Toon’, ‘Black Toon’, and ‘Green Toon’—which were cultivated under identical ecological conditions and processed into leaf teas via a standardized procedure. Untargeted metabolomics integrated with chemometric analysis were applied to systematically characterize the cultivar-dependent metabolite profiles and identify characteristic differential components. Meanwhile, a high-fat-diet-fed mouse model was used to evaluate and compare the in vivo regulatory effects on lipid metabolism, including serum lipid profiles, hepatic lipid deposition, and adipose tissue morphology. This study aimed to investigate the influences of cultivar variation on the bioactive components and lipid-modulating effects of *T. sinensis* tea in a mouse model, with the goal of generating hypothesis-generating evidence that may inform elite cultivar breeding and the development of functional teas.

## 2. Materials and Methods

### 2.1. Chemicals

Quercetin, kaempferol, rutin, and gallic acid reference standards were supplied by Chem Faces (Wuhan, China). Analytical-grade methanol and formic acid were purchased from Sigma-Aldrich (St. Louis, MO, USA). Kits for measuring serum triglycerides (TG), total cholesterol (T-CHO), and low-density lipoprotein cholesterol (LDL-C) came from Nanjing Jiancheng Bioengineering Institute (Nanjing, China). Hematoxylin–eosin (H&E) and Oil Red O (ORO) staining kits were obtained from Boster Bio (Wuhan, China). All other chemicals used met analytical grade or the highest commercially available purity standards.

### 2.2. Manufacturing of Toona sinensis Leaf Tea

Three *T. sinensis* cultivars—‘Qingyouchun’ (Green Toon), ‘Hongyouchun’ (Red Toon), and ‘Heiyouchun’ (Black Toon)—were grown in adjacent plots within the same nursery (33.13° N, 115.66° E, Fuyang, Anhui, China) under identical soil, irrigation, fertilization, and pest management conditions. Leaf samples were harvested in May by selecting one bud with 5–7 tender leaves per shoot. Five independent biological replicates were collected, each comprising leaves pooled from three individual trees. From each tree, ~0.5 kg of leaves was harvested, which yielded a combined total of 7.5 kg of raw material for subsequent processing. Each of the five biological replicates was independently subjected to the entire procedure—tea processing, extraction, and LC-MS analysis—as a separate batch.

**Figure 1:**
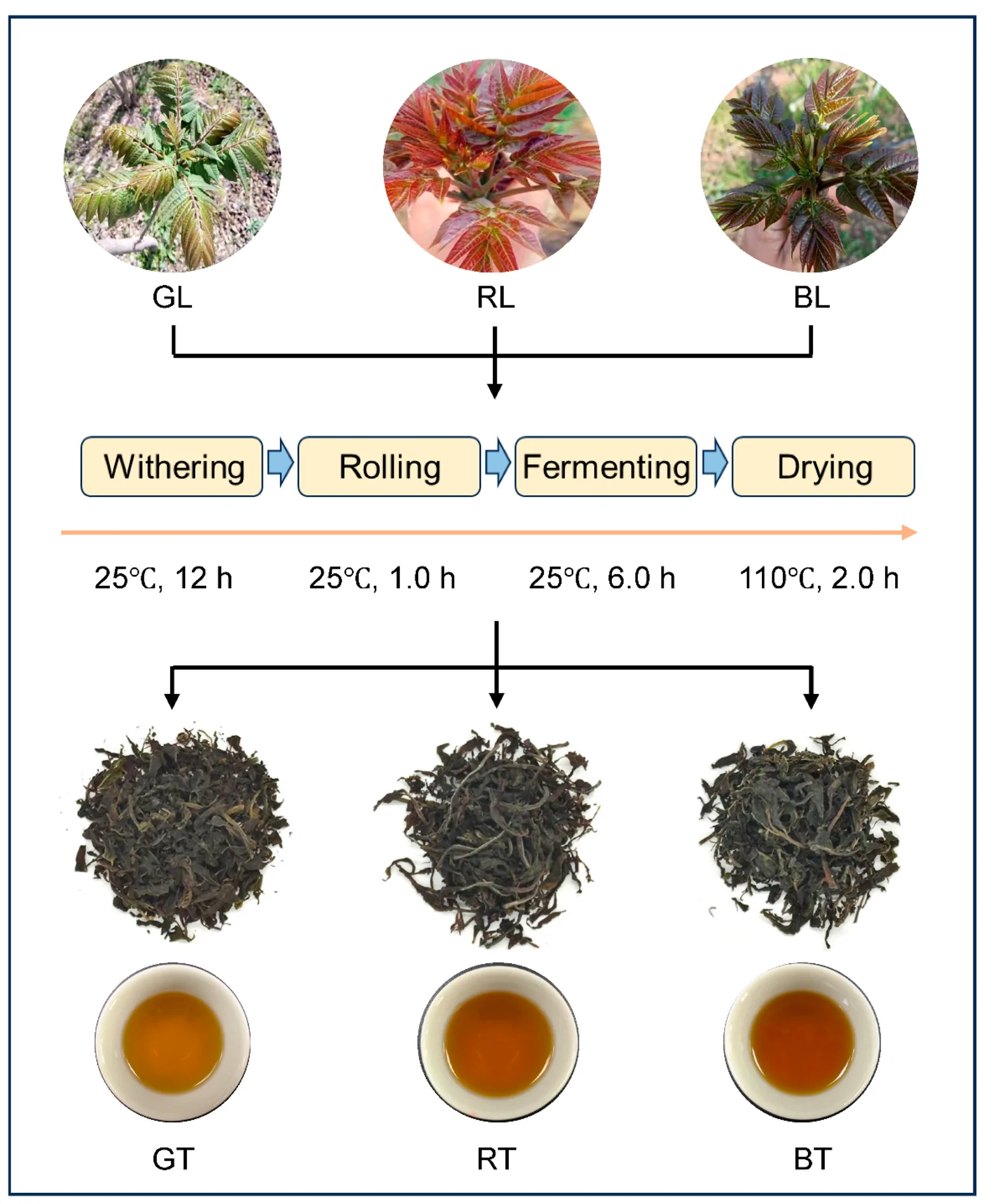
Schematic diagram of the processing procedures for the three varieties of *Toona sinensis* leaf tea. Abbreviations: GL, fresh leaves of Green Toon; RL, fresh leaves of Red Toon; BL, fresh leaves of Black Toon; GT, processed tea from Green Toon; RT, processed tea from Red Toon; BT, processed tea from Black Toon.

Tea manufacturing was performed according to a previously reported protocol [5]. The manufacturing process is shown in Figure 1. Briefly, harvested leaves were first subjected to indoor withering for 12 h, followed by 1 h of rolling to facilitate the release of flavor-active compounds. Fermentation was conducted at 25 ± 1 °C with 85 ± 5% relative humidity for 6 h, during which leaves were gently turned every 2 h to ensure uniform oxidation. Post-fermentation, leaves were dried at 110 °C for 2 h to terminate enzymatic reactions and stabilize quality, then cooled to room temperature for 30 min. The resulting teas were designated as GT, RT, and BT, respectively. Upon processing, all samples were promptly frozen and kept at −20 °C prior to subsequent analysis.

Prior to chemical analysis, a 100 g portion of each frozen sample was pulverized with a laboratory mill and then passed through a 60-mesh (250 µm) sieve to achieve homogeneous particle size distribution.

### 2.3. Sample Preparation for UHPLC-Orbitrap-MS Analysis

Tissue aliquots (100 mg) were frozen in liquid nitrogen, pulverized, and then re-suspended in pre-chilled 80% methanol followed by vortexing. After a 5-min ice incubation, the mixtures were centrifuged at 15,000× *g* and 4 °C for 20 min. The resulting supernatants were adjusted to 53% methanol using LC-MS grade water, transferred to new tubes, and re-centrifuged under identical conditions. The final supernatants were collected for LC-MS/MS analysis.

UHPLC-MS/MS was conducted on a Vanquish UHPLC system (Thermo Fisher Scientific, Germering, Germany) interfaced with an Orbitrap Q Exactive™ HF mass spectrometer (Thermo Fisher Scientific, Bremen, Germany) [5]. Chromatographic separation employed a Hypersil Gold column (100 × 2.1 mm, 1.9 μm) at a flow rate of 0.2 mL/min using a 12-min linear gradient. Mobile phases consisted of A (0.1% formic acid in water) and B (methanol). The gradient profile was as follows: 2% B from 0–1.5 min, ramped to 85% B from 1.5–4.5 min, then to 100% B from 4.5–11.5 min, followed by a return to 2% B by 12 min. MS settings included: spray voltage 3.5 kV, capillary temperature 320 °C, sheath gas 35 psi, auxiliary gas 10 L/min, S-lens RF 60, and auxiliary gas heater at 350 °C. Overlaid TIC chromatograms of QC samples are presented in Figure S1.

### 2.4. Data Processing and Analysis

Raw data were processed using XCMS for peak detection, alignment, and quantification. Key parameters included a mass tolerance of 5 ppm, a signal intensity tolerance of 30%, and QC-based peak area correction. Metabolites with CV > 30% in QCs were excluded. Metabolite annotation was performed against Novogene’s in-house MS/MS library (NovoMetDB, Novogene Co., Ltd., Beijing, China), which integrates spectra from authentic standards and curated public databases (HMDB, RefMetaDB, ReSpect). All annotated metabolites were assigned MSI confidence levels. PCA and PLS-DA were conducted using MetaboAnalyst 5.0 [16]. The PLS-DA models were validated by 200 permutation tests to assess the risk of overfitting. Differential metabolites were defined by VIP > 1, *p* < 0.05, and |fold change| ≥ 1.5.

### 2.5. Quantification of Quercetin and Kaempferol Among TLTs

Key metabolites were extracted under optimized conditions, as established based on previous studies [20] and our preliminary optimization experiments. In brief, 100 mg of toon leaf tea powder underwent two rounds of extraction using 8.0 mL of formic acid/methanol (5:95, *v*/*v*) in a 60 °C water bath, each for 30 min. After centrifugation at 3500 rpm for 10 min, the pooled supernatants were brought to a final volume of 10.0 mL with the same solvent, passed through a 0.22 µm membrane filter, and then analyzed by UPLC. Detailed UPLC operating parameters are provided in “Methods S1” of the Supporting Information. Representative chromatograms of quercetin and kaempferol are shown in Figure S2. Calibration curves for all reference standards showed excellent linearity (R^2^ > 0.998) within the tested concentration ranges, as summarized in Table S1.

### 2.6. Quantification of Major Bioactive Components in TLTs Infusions

GT, RT, and BT infusions were prepared at 20 mg/mL (1 g dried tea/50 mL water). The main bioactive components and chemical compositions in these infusions were quantified using validated standard methods. Specifically, total flavonoid content was assessed via aluminum nitrate colorimetry, with rutin serving as the reference standard and detection at 510 nm [21]. Total phenolics were determined using the Folin–Ciocalteu method, employing gallic acid as the standard and measuring absorbance at 765 nm [22]. Soluble protein levels were measured with the Bradford assay, standardized against BSA (λ = 595 nm). Free amino acid content was analyzed via the ninhydrin reaction, with glycine as the standard (λ = 570 nm) [23]. All spectrophotometric readings were conducted in triplicate on a Shimadzu UV-2600 spectrophotometer (Shimadzu Corporation, Kyoto, Japan), with appropriate blank subtractions applied. Calibration curve details are provided in Table S2.

### 2.7. Animal Experimental Design and Treatment

Five-week-old male C57BL/6J mice were purchased from Ziyuan Co., Ltd. (Hangzhou, China; SCXK2024-0004) and acclimated for 1 week under specific pathogen-free (SPF) conditions at the Animal Facility Center of Fuyang Normal University. Mice were then randomly assigned to six groups (*n* = 8 per group) using a computer-generated random number table: low-fat diet control (CK), high-fat diet (HFD), three HFD groups supplemented with Green, Red, or Black toon tea infusions (GT, RT, and BT, respectively), and HFD plus orlistat as a positive control (PC, 20 mg/kg). The sample size was based on previous HFD studies [24,25] using n = 6–8 per group, the 3R principles, and a post hoc power analysis (α = 0.05, power > 0.80). GT, RT, and BT tea infusions (20 mg/mL, prepared as described in Section 2.6) were administered via drinking water. Based on an average mouse weight of ~30 g and daily fluid intake of 4~5 mL, the corresponding dose was 2.67 mg/g body weight. Allometric scaling to a 60-kg human equated to 14 g tea/day (~4–6 cups), representing the higher end of typical human consumption. Low-fat (12% energy from fat, 3.6 kcal/g; Table S3) and high-fat (60% energy from fat, 5.2 kcal/g) diets were obtained from Trophic Animal Feed High-Tech Co., Ltd. (Nantong, China). Food intake and water consumption were monitored daily, and body weight was measured weekly. The tea infusion for animal treatment was prepared from the same pooled material that was used for metabolomic analysis. All mice were euthanized at week 12 of the intervention. All animal experiments were conducted in accordance with the ARRIVE guidelines and the National Institutes of Health Guide for the Care and Use of Laboratory Animals. The Institutional Animal Care and Use Committee of Fuyang Normal University approved all animal experiments (approval code: FYNU2025AC021).

### 2.8. Serum Biochemical Parameters Measurement

Serum levels of triglycerides (TG), total cholesterol (T-CHO), and low-density lipoprotein cholesterol (LDL-C) were quantified using commercial kits, with optical density (OD) values measured by a SpectraMax microplate reader (Molecular Devices, San Jose, CA, USA).

### 2.9. Histological Analysis

Liver and adipose tissues were paraffin-embedded, sectioned at 5 μm, and stained with hematoxylin–eosin (H&E) as previously described [26]. Images were captured using a LEICA DM500 microscope equipped with a LEICA ICC50W camera (Wetzlar, Germany). For quantitative analysis, histological slides were coded with random numbers, and five random fields per section were selected. Image acquisition and quantification were performed by an investigator blinded to group allocation. The proportion of normal hepatocytes and the area (μm^2^) of white adipocytes were measured using ImageJ 2.14.0 software (NIH, Bethesda, MD, USA) following established protocols [27].

### 2.10. Statistical Analysis

Data are presented as mean ± SEM. Body weight curves were analyzed using two-way repeated-measures ANOVA (treatment × time), with Greenhouse–Geisser correction when sphericity was violated, followed by Tukey’s post hoc test. Other endpoints were analyzed by unpaired *t*-test or one-way ANOVA followed by Tukey’s test. Statistical significance was set at *p* < 0.05.

## 3. Results

### 3.1. Significant Cultivar-Dependent Differences in Metabolite Profiles Among TLTs

**Figure 2:**
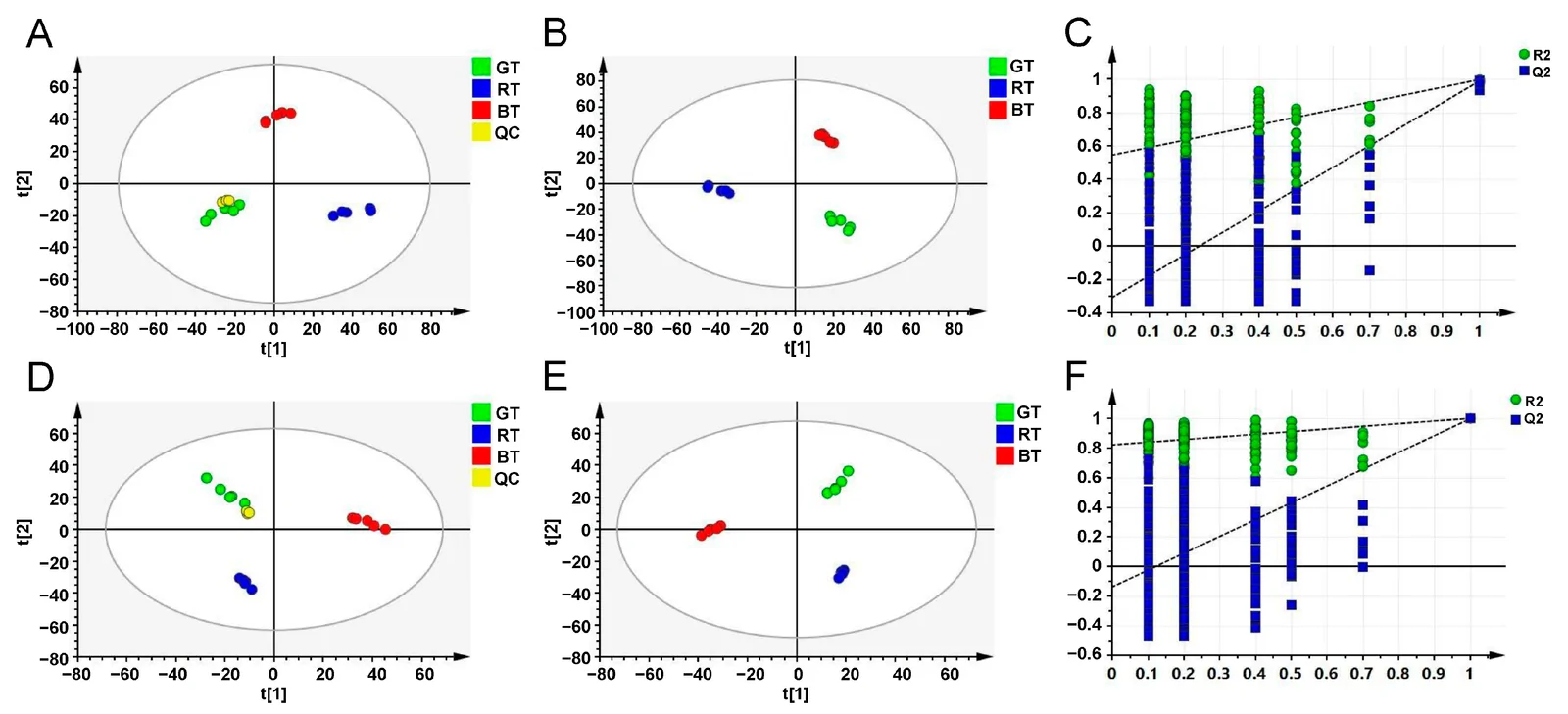
Multivariate statistical analysis of metabolite profiles among the three TLTs. (**A**,**D**) PCA score plots for positive ((**A**): R^2^X = 0.732, Q^2^ = 0.611) and negative ((**D**): R^2^X = 0.767, Q^2^ = 0.592) ionization modes. (**B**,**E**) PLS-DA score plots for positive ((**B**): R^2^X = 0.723, R^2^Y = 0.998, Q^2^ = 0.991) and negative ((**E**): R^2^X = 0.769, R^2^Y = 0.999, Q^2^ = 0.996) ionization modes. (**C**,**F**) Permutation tests for PLS-DA models in positive ((**C**): R^2^ = 0.546, Q^2^ = −0.307) and negative ((**F**): R^2^ = 0.822, Q^2^ = −0.136) ionization modes.

Teas designated as GT (Green Toon), RT (Red Toon), and BT (Black Toon) were prepared from ‘Qingyouchun’, ‘Hongyouchun’, and ‘Heiyouchun’ cultivars of *T. sinensis*, respectively, following a unified processing method (Figure 1). Subsequently, the chemical profiles of these three TLTs were analyzed using UHPLC-Orbitrap-MS [5]. Positive and negative ion modes yielded 2547 and 1929 ion features, respectively. Principal component analysis (PCA) was first performed to assess global metabolic differences. In positive ion mode, GT, RT, and BT samples clustered predominantly in the third, first/second, and fourth quadrants, respectively (Figure 2A). In negative ion mode, GT samples clustered in the second quadrant, RT samples in the third, and BT samples in the first and fourth quadrants (Figure 2D). The tight clustering of qualimetabolomicssamples confirmed the stability and reliability of the metabolomics analysis. These results clearly indicate that the three TLTs possess distinct and cultivar-dependent chemical profiles.

To further validate these differences, supervised partial least squares discriminant analysis (PLS-DA) was conducted [16]. In positive ion mode, PLS-DA clearly separated the three groups along the second principal component, with GT, RT, and BT samples distinctly separated from each other (Figure 2B). In negative ion mode, GT, RT, and BT samples also formed three distinct clusters with clear separation (Figure 2E). The model parameters (R^2^X, R^2^Y, and Q^2^) were 0.723, 0.998, and 0.991 in positive ion mode, and 0.769, 0.999, and 0.996 in negative ion mode, indicating robust model performance. Permutation tests confirmed the absence of overfitting in either ion mode (Figure 2C,F).

### 3.2. Comparative Analysis of Differential Metabolites Among TLTs

**Figure 3:**
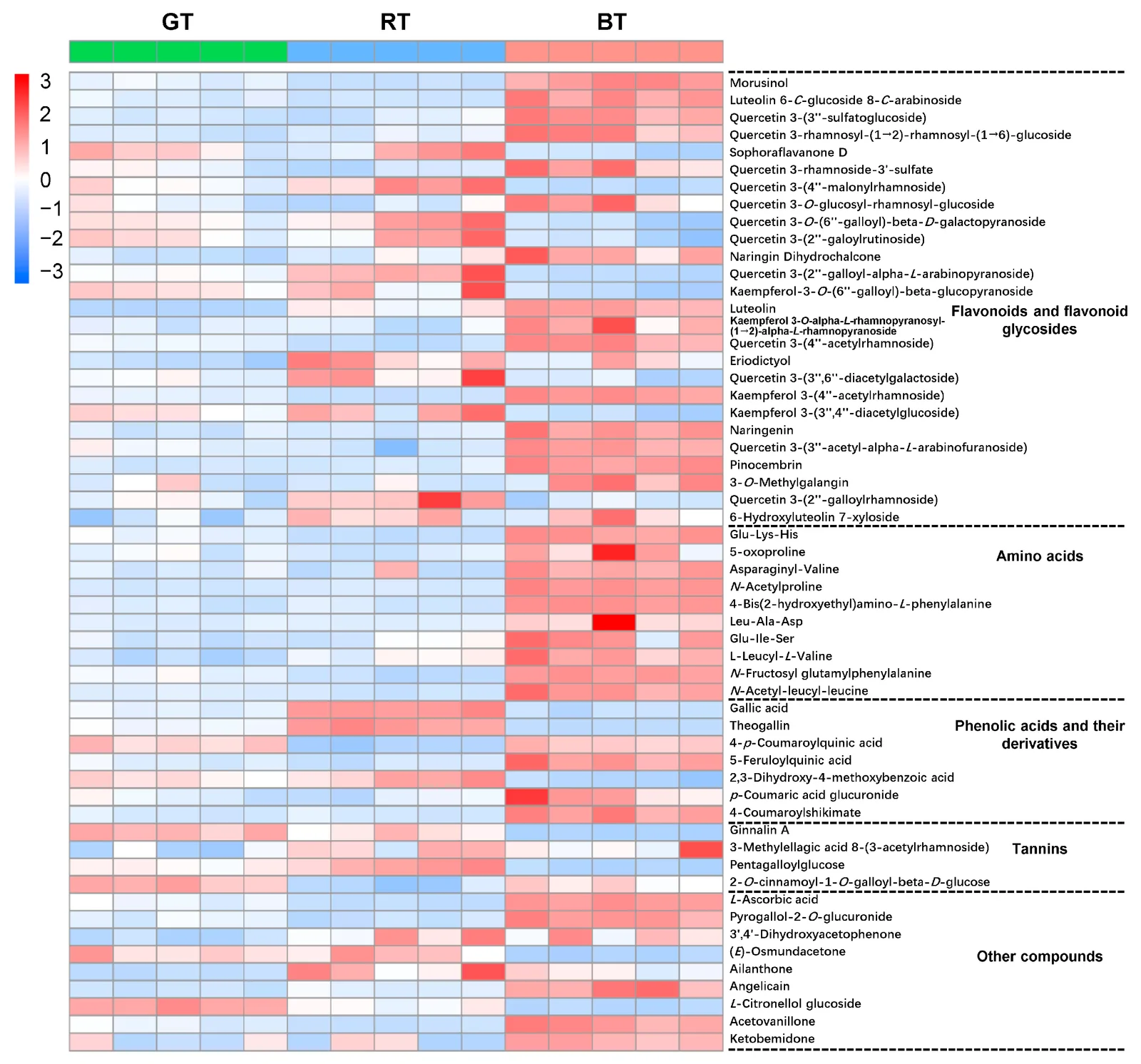
Heatmap of differential metabolites identified in TLTs.

Using criteria of VIP > 1, *p* < 0.05, and |fold change| > 1.5, a total of 56 differential metabolites were putatively annotated across TLTs by retention time, *m/z*, MS/MS fragments, database matching, and literature comparison. These comprised 26 flavonoids and flavonoid glycosides, 10 amino acids, 7 phenolic acids and their derivatives, 4 tannins, and 9 other compounds (Table 2). The MSI confidence levels and diagnostic MS/MS fragments for all 56 differential metabolites are provided in Supplementary Table S4. A heatmap was generated to visualize the abundance patterns of these metabolites among the three varieties (Figure 3).

#### 3.2.1. Flavonoids and Flavonoid Glycosides

Flavonoids and their glycosides are key bioactive constituents in toona. The relative abundances of 26 such compounds varied significantly among TLTs (Table 2). Black Toon (BT) exhibited the highest levels of most flavonoid components, followed by Red Toon (RT), while Green Toon (GT) showed the lowest overall abundance. BT was putatively characterized by a remarkable enrichment of flavonoid sulfates. Although RT and GT shared some similarities in overall metabolic profiles, RT contained significantly higher levels of most putatively identified acylated flavonoid glycosides. In particular, the abundance of quercetin 3-(4″-malonylrhamnoside) in RT was 3.51-fold that in BT, and the contents of several flavonoid compounds in RT were even comparable to or higher than those in BT. These results indicate distinct divergence in flavonoid metabolic pathways and modification patterns among the three varieties, suggesting that RT and BT possess greater potential for applications in functional foods targeting lipid metabolism regulation.

**Figure 4:**
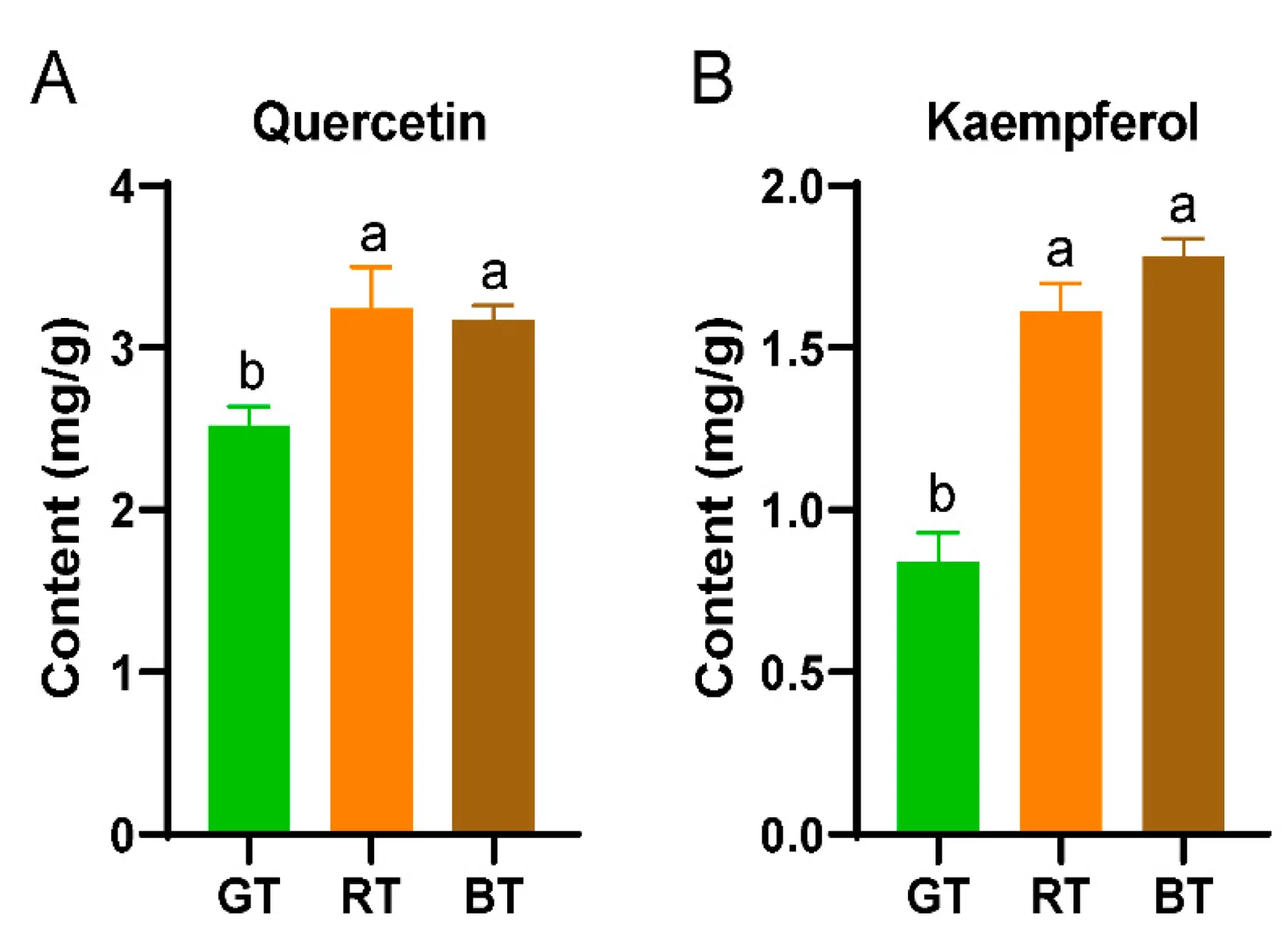
Quantification of quercetin and kaempferol among TLTs. Columns with different letters are significantly different (*p* < 0.05). Values are presented as means ± SEM (n = 3).

Untargeted metabolomics revealed significantly higher relative abundances of flavonoids in RT and BT compared to GT. Subsequent absolute quantification using UPLC with authentic standards confirmed that quercetin and kaempferol contents were significantly higher in RT and BT (Figure 4), consistent with the metabolomic profiling results.

#### 3.2.2. Amino Acids

Amino acids play a critical role in the umami taste and aroma development of *Toona* shoots. Analysis of ten amino acid-related metabolites revealed that BT exhibited the highest overall relative abundances, whereas GT and RT showed comparable abundances with no substantial differences across most components. Only a few dipeptides and tripeptides displayed minor variations, with no clear directional bias between GT and RT.

#### 3.2.3. Phenolic Acids and Their Derivatives

Seven phenolic acids and their derivatives were differentially accumulated. RT exhibited pronounced accumulation of gallic acid, theogallin, and 2,3-dihydroxy-4-methoxybenzoic acid, with theogallin relative abundance reaching 6.48 times that of BT, representing a distinct metabolic feature. In contrast, chlorogenic acid derivatives (4-p-coumaroylquinic acid, 5-feruloylquinic acid) and coumaric acid derivatives (p-coumaric acid glucuronide, 4-coumaroylshikimate) were most abundant in BT, with RT showing slightly lower levels. GT generally displayed intermediate levels across most indices.

#### 3.2.4. Tannins

Four tannin compounds exhibited differential accumulation among TLTs. Ginnalin A was most abundant in GT, with a level 3.06 times that of BT, followed by RT. Pentagalloylglucose accumulated predominantly in RT, reaching 3.38 times the BT level. 2-*O*-Cinnamoyl-1-*O*-galloyl-β-*D*-glucose was higher in GT than in RT and BT, whereas 3-methylellagic acid 8-(3-acetylrhamnoside) was slightly elevated in RT compared to BT and lowest in GT. Collectively, these results highlight substantial intervarietal differences in tannin metabolism, with characteristic tannins in GT and RT significantly exceeding those in BT.

#### 3.2.5. Other Compounds

Nine additional differential metabolites were putatively annotated, including L-ascorbic acid, pyrogallol-2-*O*-glucuronide, 3′,4′-dihydroxyacetophenone, (*E*)-osmundacetone, ailanthone, angelicain, L-citronellol glucoside, acetovanillone, and ketobemidone. BT displayed the highest relative abundances of most of these compounds, including ascorbic acid, pyrogallol-2-*O*-glucuronide, angelicain, acetovanillone, and ketobemidone, while GT and RT generally showed lower abundances. (*E*)-osmundacetone was significantly enriched in both GT and RT, with levels approximately 2.5 times those in BT. Ailanthone and 3′,4′-dihydroxyacetophenone were notably higher in RT, whereas L-citronellol glucoside was most abundant in GT.

### 3.3. Quantification of Major Bioactive Components in Tea Infusions of TLTs

**Figure 5:**
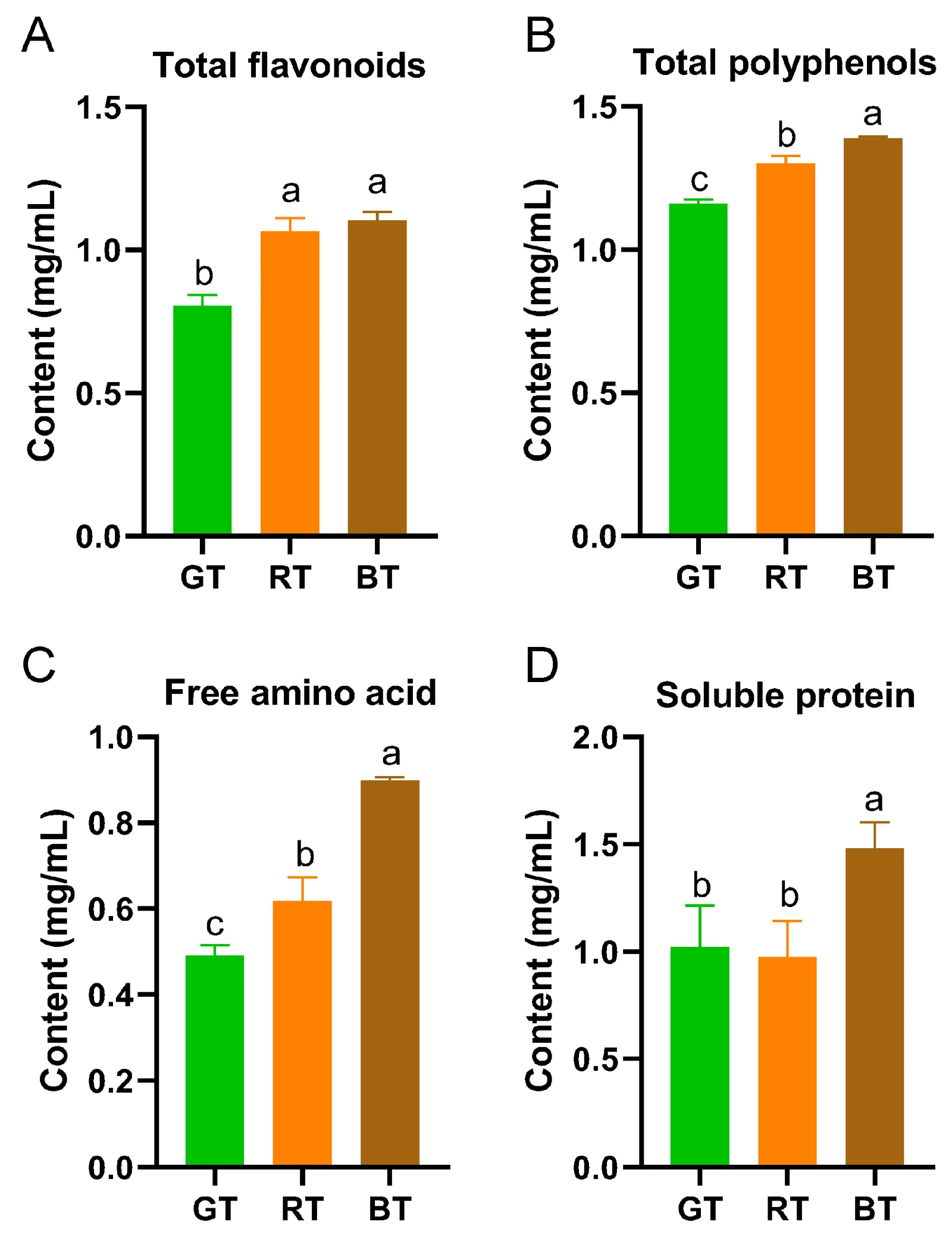
Quantification of major bioactive components in TLTs infusions. Columns with different letters are significantly different (*p* < 0.05). Values are presented as means ± SEM (n = 3).

Significant differences in the contents of total flavonoids, total polyphenols, free amino acids, and soluble protein were observed among the three TLT infusions (Figure 5). Compared to GT, both RT and BT contained significantly higher levels of total flavonoids (RT: 1.07 ± 0.03, BT: 1.10 ± 0.02, GT: 0.80 ± 0.02 mg/mL; *p* < 0.05) and total polyphenols (RT: 1.30 ± 0.01, BT: 1.39 ± 0.01, GT: 1.16 ± 0.01 mg/mL; *p* < 0.05). Notably, BT exhibited the highest polyphenol content among the three varieties (*p* < 0.05 vs. GT and RT). A similar trend was observed for free amino acids: RT (0.62 ± 0.03 mg/mL) and BT (0.90 ± 0.01 mg/mL) both had significantly higher contents than GT (0.49 ± 0.01 mg/mL; *p* < 0.05), with BT also showing markedly higher levels than RT (*p* < 0.01). For soluble protein, BT exhibited the highest content (1.48 ± 0.07 mg/mL), which was significantly greater than that of both GT (1.02 ± 0.11 mg/mL) and RT (0.97 ± 0.09 mg/mL; *p* < 0.05), while no significant difference was found between GT and RT.

### 3.4. Effects of TLTs on Lipid Metabolism Parameters in HFD-Fed Mice

**Figure 6:**
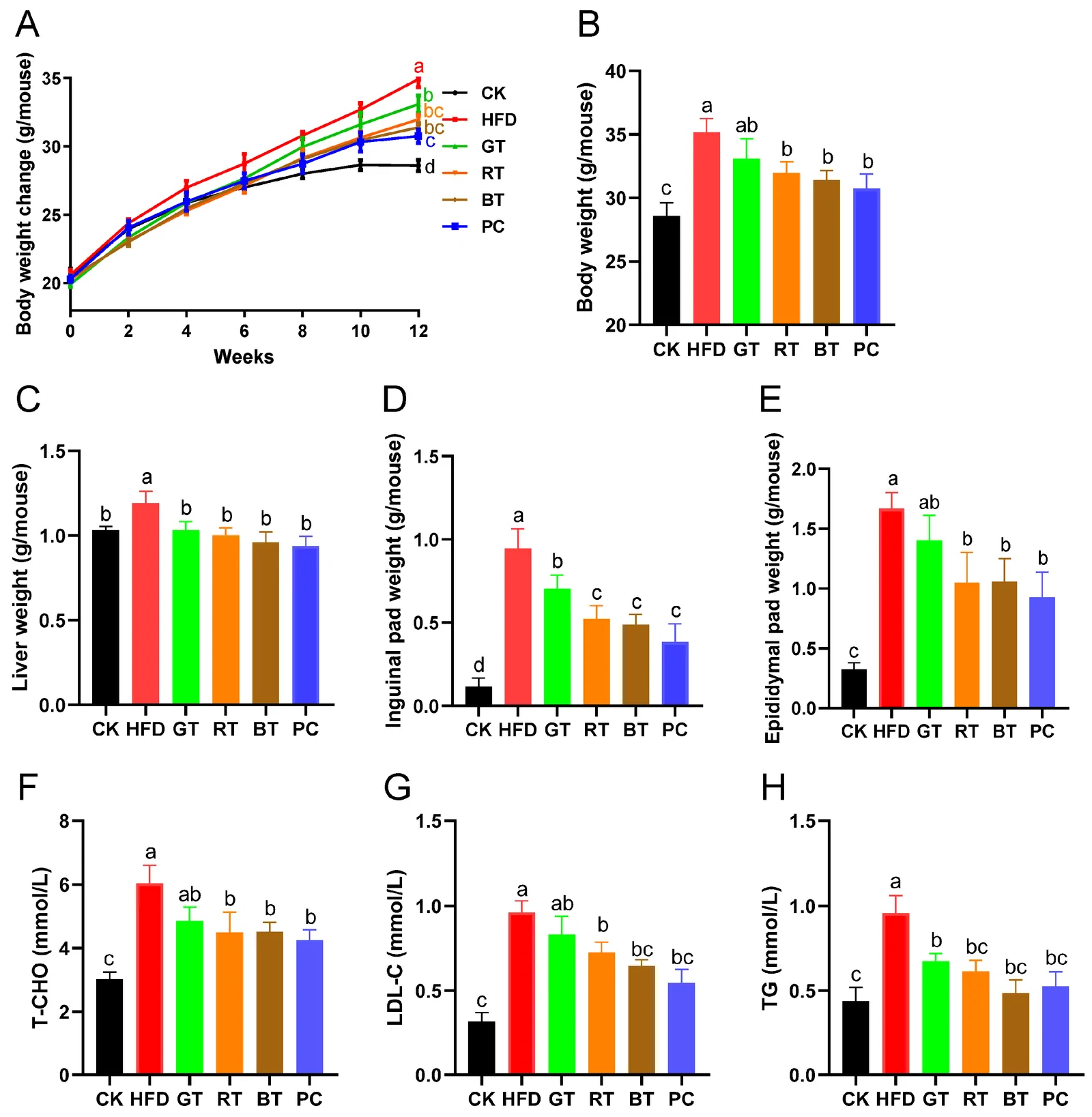
Effects of TLTs on lipid metabolism parameters in HFD-induced obese mice. (**A**) Body weight curves; (**B**) body weight; (**C**) liver weight; (**D**) inguinal fat pad weight; (**E**) epididymal fat pad weight; (**F**) serum total cholesterol; (**G**) serum low-density lipoprotein cholesterol; (**H**) serum triglycerides. Columns with different letters are significantly different (*p* < 0.05). Values are presented as means ± SEM (n = 8).

To evaluate the effects of TLTs on lipid metabolism in the context of high-fat diet feeding, HFD-fed mice were orally administered TLTs for 12 consecutive weeks. As shown in Figure 6A, HFD feeding resulted in a marked increase in body weight. Following 12 weeks of treatment, both TLTs and the positive control Orlistat attenuated this weight gain as well as other HFD-induced changes. Notably, treatment with RT, BT, or Orlistat significantly reduced body weight, liver weight, inguinal fat pad weight, and epididymal fat pad weight, whereas GT treatment only significantly decreased liver and inguinal fat pad weights (Figure 6B–E).

To assess the effects on circulating lipid levels, serum concentrations of T-CHO, LDL-C, and TG were measured. After 12 weeks, the HFD group exhibited significantly elevated levels of T-CHO, LDL-C, and TG compared with the control (CK) group (Figure 6F–H), confirming the establishment of diet-induced hyperlipidemia. Treatment with RT, BT, or Orlistat significantly improved all three lipid parameters, whereas GT treatment only significantly lowered TG levels.

To determine whether the observed effects on lipid metabolism parameters were attributable to changes in food intake, food and water consumption were monitored throughout the experiment. No significant differences in food intake were observed between the HFD group and any of the TLT-treated groups, although the HFD group consumed more food than the CK group (Figure S3A). Similarly, water consumption did not differ significantly among any of the groups (Figure S3B).

### 3.5. TLTs Ameliorate Hepatic Steatosis and Adipocyte Hypertrophy in HFD-Induced Obese Mice

**Figure 7:**
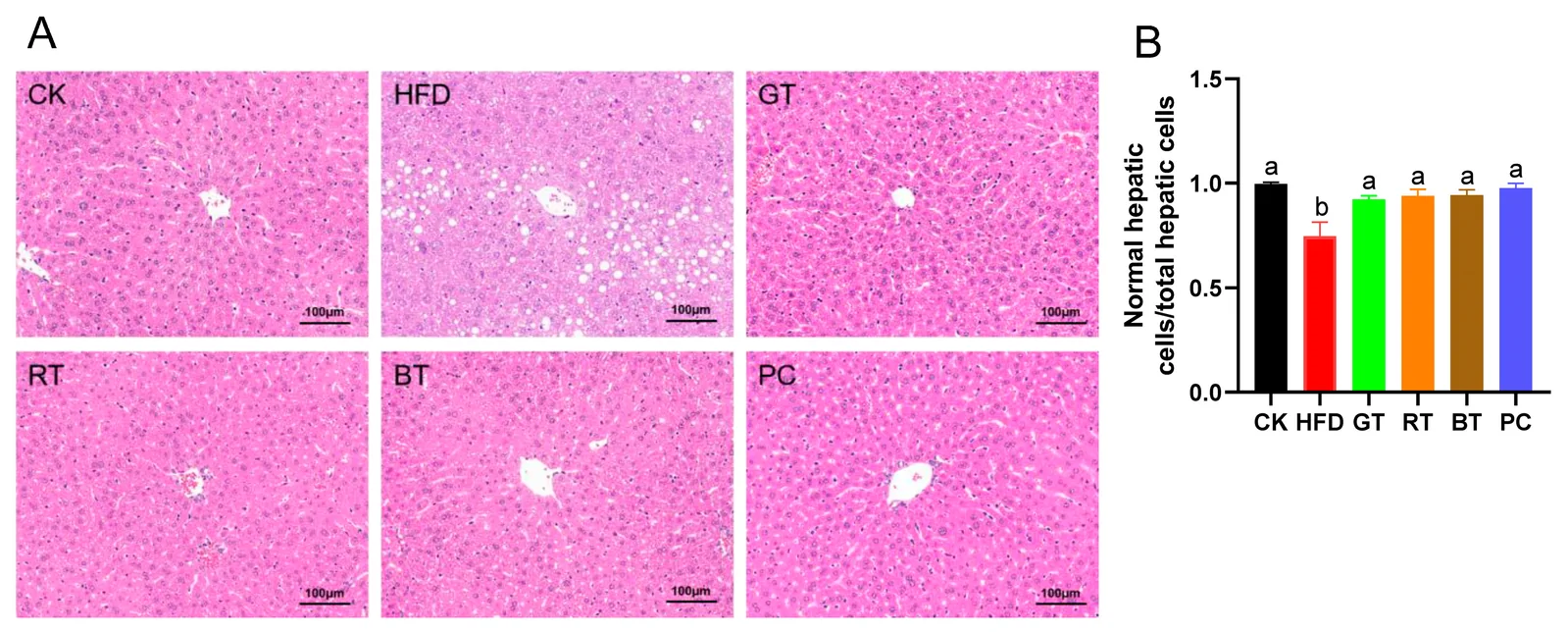
TLTs ameliorate hepatic steatosis in HFD-induced obese mice. (**A**) Representative H&E-stained liver sections; (**B**) quantitative analysis of normal hepatocyte ratio. Columns with different letters are significantly different (*p* < 0.05).

The liver serves as a central organ in systemic lipid metabolism, and its histological changes were evaluated by H&E staining. In the CK group, liver sections showed normal polygonal hepatocytes with clear round nuclei and well-preserved hepatic lobular architecture (Figure 7A). In sharp contrast, mice in the HFD group developed severe hepatic steatosis, characterized by diffuse macrovesicular and microvesicular fatty infiltration, disrupted hepatic lobule structure, and the presence of inflammatory foci (Figure 7A). Treatment with TLTs or orlistat markedly ameliorated these pathological histological features; quantitative analysis indicated that the proportion of normal hepatocytes was significantly higher in the GT-, RT-, BT-, and orlistat-treated groups compared with the HFD group (Figure 7B, *p* < 0.05).

**Figure 8:**
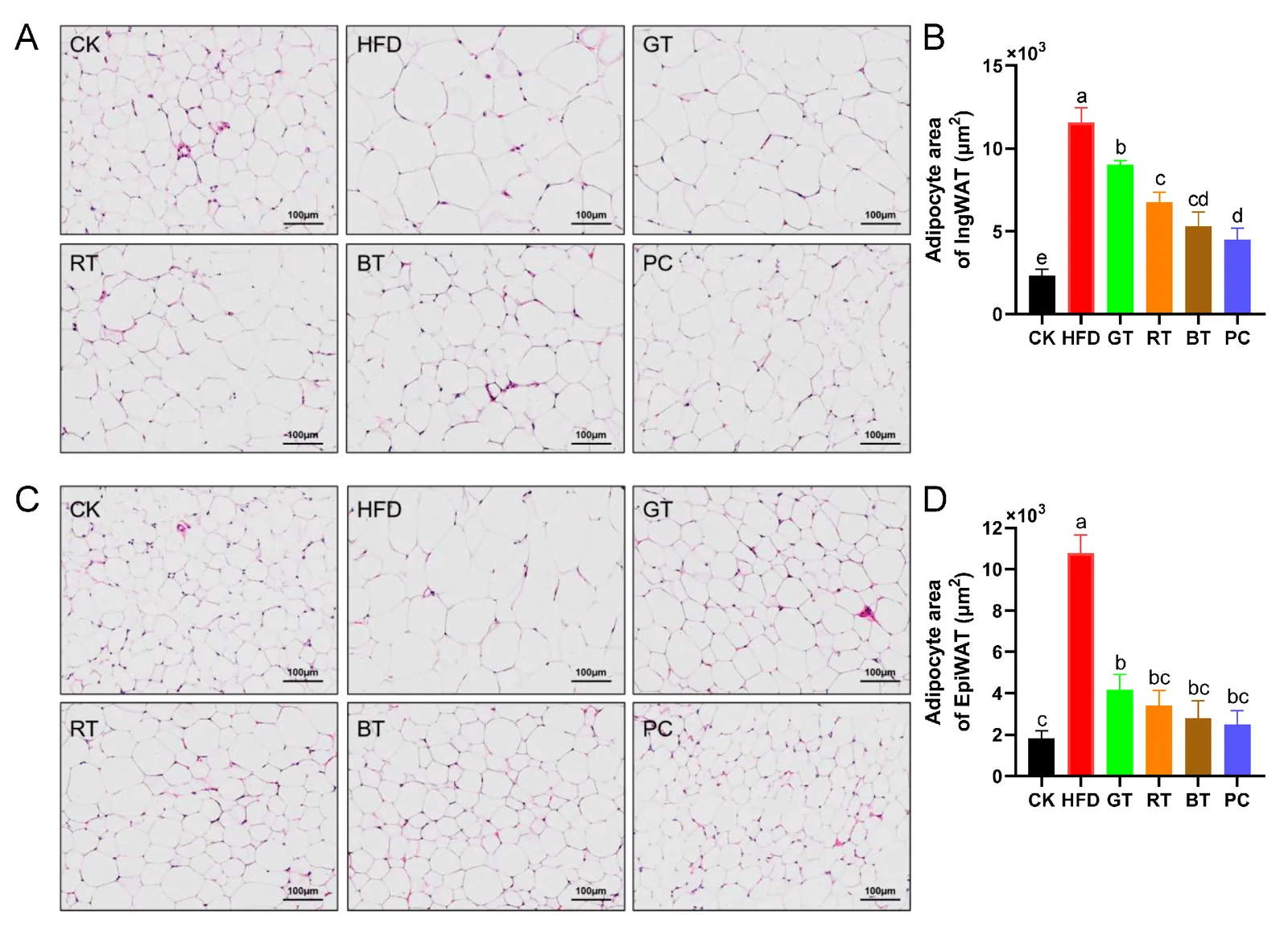
TLTs attenuate adipocyte hypertrophy in HFD-induced obese mice. (**A**) H&E staining of inguinal white adipose tissue (ingWAT); (**B**) inguinal adipocyte area; (**C**) H&E staining of epididymal white adipose tissue (epiWAT); (**D**) epididymal adipocyte area. Columns with different letters are significantly different (*p* < 0.05).

H&E staining of inguinal white adipose tissue (ingWAT) and epididymal white adipose tissue (epiWAT) further confirmed that HFD feeding induced pronounced adipocyte hypertrophy, which was reflected by a significant increase in adipocyte cross-sectional areas (Figure 8A,C). This HFD-induced hypertrophic effect was significantly attenuated by treatment with TLTs or orlistat (Figure 8B,D). Among the three TLT groups, RT and BT exhibited more potent inhibitory effects on adipocyte hypertrophy than GT, which was consistent with their more remarkable regulatory effects on fat pad weights and serum lipid profiles observed in the previous experiments.

### 3.6. Correlation Analysis Between Candidate Metabolites of TLTs and Lipid Metabolism Indicators

**Figure 9:**
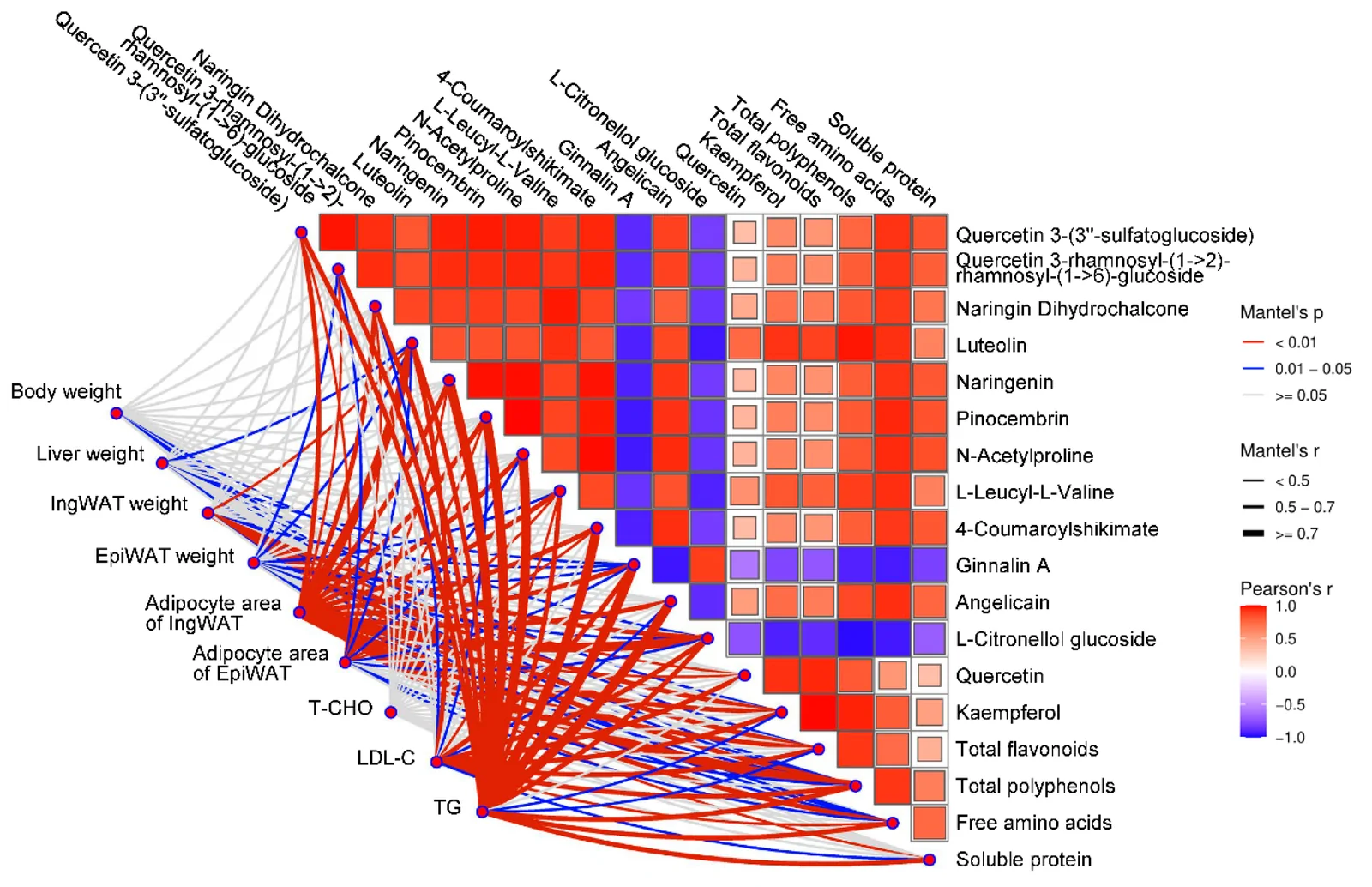
Exploratory heatmap depicting correlation analysis between candidate metabolites of TLTs and lipid metabolism parameters. Note: This heatmap is intended for descriptive visualization of association patterns only. No formal statistical inference should be derived from this figure, and the observed patterns are hypothesis-generating rather than confirmatory.

To clarify the relationship between the main candidate metabolites in TLTs and the improvements in lipid metabolism indicators, Pearson correlation analysis was performed to assess the relationships between the relative abundances of all 56 differential metabolites and lipid metabolism parameters. Of these, 18 components showed notable co-variation patterns with lipid metabolism-related parameters (Figure 9 and Table S5). Polyphenols and flavonoids, including quercetin derivatives, luteolin, and kaempferol, together with *N*-acetylproline and certain dipeptides, exhibited negative association trends with fat accumulation and serum lipid levels. In contrast, ginnalin A and *L*-citronellol glucoside displayed positive co-variation with body weight-related and lipid-related parameters, although these associations should be interpreted with caution. Notably, the combined parameters (total polyphenols, total flavonoids, and free amino acids) showed stronger negative association trends than most individual compounds, suggesting potential additive or synergistic effects (Figure 9). Because this analysis is based on cultivar-level mean values (n = 3 per cultivar), the observed patterns are descriptive and hypothesis-generating; they are not intended to establish statistical significance or causality, and the annotated candidate metabolites require further validation using targeted approaches.

## 4. Discussion

The health benefits of *T. sinensis* are well recognized, yet how cultivar variation influences its metabolite profiles and lipid-modulating activities remains poorly understood. In this study, we systematically characterized cultivar-dependent metabolic signatures of leaf teas prepared from three *T. sinensis* cultivars (GT, RT, BT) using an integrated strategy of metabolomics, phytochemical quantification, and a 12-week high-fat diet (HFD) mouse model. Our results demonstrate that cultivar strongly shapes the metabolomic composition of *T. sinensis* leaf teas (TLTs), which in turn leads to distinct efficacies in alleviating HFD-induced metabolic disorders. Specifically, RT and BT exhibited specialized metabolite profiles and superior lipid-lowering bioactivities, whereas GT showed a less favorable phytochemical profile and weaker protective effects. These findings suggest a potential chemotype–bioactivity association and offer preliminary evidence that may guide cultivar selection for further development of functional teas targeting metabolic health.

Notably, because the tea infusions were prepared at equal dry mass, the higher contents of total flavonoids, polyphenols, and free amino acids in RT and BT (Figure 5) mean that, at the same administered volume, mice receiving RT or BT ingested a higher load of these candidate bioactive constituents compared with GT. This may partly explain the superior efficacy of RT and BT observed in the HFD-fed mice.

It should be noted that our research group has conducted previous studies on *T. sinensis* leaf tea. We previously reported the effects of processing methods (direct drying vs. black tea processing) on the metabolite signatures and hypoglycemic effects of the ‘Black Toon’ cultivar, demonstrating that black tea processing significantly enriched flavonoids (from 26.31 to 35.48 mg/g) and free amino acids (from 42.87 to 47.36 mg/g) [5]. Subsequently, we compared the impact of harvest maturity (tender leaves vs. mature leaves) and found that tender leaves had higher levels of total polyphenols (26.73 mg/g vs. 18.03 mg/g) and total flavonoids (35.68 mg/g vs. 26.31 mg/g) than mature leaves, with quercetin and kaempferol derivatives being the predominant flavonoids [16]. However, both of these studies were limited to a single cultivar and thus failed to address the critical issue of cultivar variation. The present study, for the first time, introduces the cultivar dimension into the research framework of *T. sinensis* leaf tea, systematically comparing three dominant cultivars (‘Green Toon’, ‘Red Toon’, and ‘Black Toon’) under identical black tea processing conditions with respect to their metabolite profiles and lipid metabolism-regulating effects. Therefore, this work is not a mere extension of previous studies but rather fills a critical gap in the functional evaluation of *T. sinensis* leaf tea from the novel perspective of cultivar variation, providing preliminary scientific evidence for elite cultivar screening.

PCA and PLS-DA revealed clear and robust separation among the three TLTs, confirming prominent cultivar-dependent divergence in phytochemical composition. The most pronounced differences were observed in flavonoid abundance and structural modification. RT and BT showed significantly higher relative abundances of quercetin- and kaempferol-derived flavonoids. These compounds have been reported in previous studies to modulate lipid homeostasis via activating AMPK, suppressing SREBP-1c, and inhibiting PPARγ-mediated adipogenesis [28,29,30,31]. In our exploratory analysis, these flavonoids showed negative co-variation trends with serum lipid parameters (Figure 9), further supporting their role as key bioactive contributors. Notably, RT was distinguished by putative high relative abundances of acylated flavonoid glycosides, especially the putatively annotated malonylated quercetin derivatives such as quercetin 3-(4″-malonylrhamnoside) (3.51-fold higher than in BT), which, if confirmed, can enhance compound stability and serve as a prodrug system for sustained quercetin release [32]. BT, by contrast, was putatively enriched in flavonoid sulfates, which may improve aqueous solubility and systemic bioavailability [33]. These structural modifications likely contribute to the comprehensive lipid-lowering effects of RT and BT. In comparison, GT contained lower overall flavonoid levels and had lower levels of such specialized metabolites, consistent with its relatively modest bioactivity.

Beyond flavonoids, phenolic acid profiles also exhibited marked cultivar dependence. RT was highly enriched in gallic acid and theogallin (theogallin content reaching 6.48-fold that of BT), whereas BT accumulated more chlorogenic acid derivatives including 4-p-coumaroylquinic acid and 5-feruloylquinic acid. Although chlorogenic acids are recognized to benefit metabolic health [34,35], correlation analysis indicated that total polyphenol content, rather than individual phenolic compounds, showed the strongest association with improved metabolic profiles (Figure 9). This suggests that the collective activity of the polyphenol pool primarily drives the observed efficacy. Regarding amino acids, BT contained the highest level of free amino acids (0.90 ± 0.01 mg/mL in tea infusion, significantly higher than in RT and GT), which contribute favorably to sensory quality [36] but showed weaker correlations with metabolic parameters than flavonoids and polyphenols (Figure 9). Thus, amino acids primarily influence palatability rather than directly mediating metabolic regulation.

In the HFD-fed mouse model, RT and BT significantly attenuated body weight gain, liver weight, fat deposition, and adipocyte hypertrophy without altering food or water intake (Figure S3; Figure 6, Figure 7 and Figure 8), suggesting that these effects were not primarily driven by reduced energy intake. Their lipid-lowering effects (reductions in serum total cholesterol, LDL-cholesterol, and triglycerides) showed similar trends to those of orlistat under the current experimental conditions, although the underlying mechanisms are likely distinct (orlistat acts via inhibition of intestinal lipases, whereas TLTs may exert systemic regulation). These lipid-lowering effects are consistent with the mechanisms proposed for tea polyphenols and flavonoids as discussed above, although the involved pathways were not directly measured in the present study. The negative associations of *N*-acetylproline and small peptides with lipid parameters suggest that amino acid derivatives and peptides may cooperate with flavonoids in hepatic lipid clearance, but this hypothesis requires direct testing. The positive associations of ginnalin A and L-citronellol glucoside with adverse metabolic phenotypes should be interpreted cautiously, as they may reflect confounding, compensatory responses, or matrix effects rather than harmful effects. Validation using purified compounds in both in vitro and in vivo systems is required.

An important consideration is that a cultivar may contain both beneficial and potentially unfavorable metabolites. In our exploratory analysis, ginnalin A and *L*-citronellol glucoside correlated positively with adiposity parameters. However, these correlations should be interpreted cautiously because they are based on cultivar-level means, and GT—which had the highest levels of these compounds—showed the weakest lipid-lowering effects. Overall efficacy was more strongly associated with total polyphenols and flavonoids than with any single metabolite. Therefore, cultivar selection should be based on integrated chemical profiles and in vivo efficacy. RT and BT remain promising, but safety evaluation of their minor constituents is needed in future studies.

Beyond the mechanistic insights discussed above, the translational potential of TLTs warrants consideration. From a translational perspective, the daily intake of TLTs in mice (converted to human equivalent dose based on body surface area) corresponds to approximately 4–6 cups of tea per day for a 60 kg adult, supporting the potential applicability of RT and BT as dietary interventions. From a functional food standpoint, GT is more suitable for culinary use as a vegetable ingredient [37], owing to its relatively low levels of bioactive flavonoids and limited lipid-lowering efficacy. In contrast, RT and BT are elite cultivars with distinct functional advantages: RT is putatively characterized by acylated flavonoid glycosides and pentagalloylglucose, whereas BT putatively stands out for its high contents of polyphenols, flavonoid sulfates, and free amino acids. These differentiated chemical profiles enable targeted cultivar selection and precision product development for metabolic health management.

Several limitations should be acknowledged. First, although our study demonstrates strong associations between cultivar-specific metabolite profiles and lipid-modulating effects in mice, the correlative nature of these findings precludes causal inferences. Establishing direct causal relationships would require future studies using bioassay-guided fractionation, reconstitution experiments, or purified compound interventions. In addition, the lack of targeted absolute quantification for flavonoid sulfates, acylated flavonoids, and theogallin constrains the robustness of the chemotype–bioactivity correlations. Second, because whole TLT infusions were administered in vivo, the individual contributions of specific metabolites versus matrix synergism remain to be disentangled. Bioassay-guided fractionation or reconstitution experiments could help clarify causal relationships. Third, the unexpected positive correlation of ginnalin A and *L*-citronellol glucoside with adiposity requires validation using purified compounds in both in vitro and in vivo systems. Fourth, FDR correction was not applied to the *p*-values; however, the multi-criteria filtering strategy (VIP, *p*, fold change) mitigates false positives, and the annotated metabolites should therefore be considered exploratory. Finally, translation to human relevance requires controlled clinical trials to confirm whether the cultivar-dependent metabolic benefits observed in mice extend to human lipid metabolism.

## 5. Conclusions

This study provides evidence that cultivar-specific metabolic signatures in *T. sinensis* are associated with differential anti-hyperlipidemic efficacy of its leaf teas. RT and BT, putatively characterized by unique flavonoid modification profiles (acylation in RT, sulfation in BT) and high contents of bioactive polyphenols, significantly outperform GT in alleviating HFD-induced obesity, hepatic steatosis, and dyslipidemia. Flavonoids and total polyphenols emerged as candidate bioactive constituents, showing strong associations with improved lipid metabolism in this exploratory study. These findings suggest that cultivar-dependent phytochemical profiles are associated with differential lipid-modulating effects in mice, and they provide preliminary clues for the targeted selection of elite cultivars. Further studies are needed to confirm their translational potential for functional food development.

## Figures and Tables

**Table 1:** Comparison of key characteristics between *Toona sinensis* leaves and conventional tea leaves (*Camellia sinensis*).

Characteristic	*Toona sinensis* Leaves	Conventional Tea Leaves (*Camellia sinensis*)
Botanical classification	Meliaceae; *Toona sinensis*	Theaceae; *Camellia sinensis*
Leaf morphology	Pinnately compound; 8–20 leaflet pairs	Simple, alternate; serrate margins
Traditional usage	Seasonal vegetable (“tree vegetable”), medicinal and edible plant in traditional Chinese medicine	Traditional beverage, globally consumed; leaves processed into green, black, oolong, and other tea types
Major bioactive compounds	Flavonoids (rutin, quercetin, kaempferol glycosides), saponins, polysaccharides, alkaloids (caffeine low or negligible), vitamin C, minerals (K, Ca, P, Mg, Fe, Zn)	Tea polyphenols (catechins, especially EGCG), alkaloids (caffeine 2–4% dry weight; theophylline), L-theanine, polysaccharides
Characteristic taste and flavor compounds	Pungent, garlic-like aroma; volatile sulfur-containing compounds, terpenes (e.g., β-elemene, α-caryophyllene, trans-caryophyllene), aldehydes, alcohols	Non-volatile taste compounds: catechins, caffeine, L-theanine; volatile compounds: linalool, geraniol, and other terpenoids

Note: Data compiled from the published literature [1,9,10,11,12]. Abbreviations: EGCG, epigallocatechin-3-gallate.

**Table 2:** Differential metabolites in three varieties of toon leaf tea.

Compound Name	RT (min)	m/z	Molecular Weight	Ion Mode	Formula	Mass Error	FC (GT/BT)	FC (RT/BT)
**Flavonoids and flavonoid glycosides**								
Morusinol	1.422	437.1599	438.1679	Negative	C_25_H_26_O_7_	1.51269	0.35	0.15
Luteolin 6-*C*-glucoside 8-*C*-arabinoside	1.807	609.1452	610.1534	Negative	C_27_H_30_O_16_	1.49753	0.36	0.26
Quercetin 3-(3″-sulfatoglucoside)	5.364	543.0451	544.0523	Negative	C_21_H_20_O_15_S	0.16593	0.50	0.52
Quercetin 3-rhamnosyl-(1→2)-rhamnosyl-(1→6)-glucoside	5.422	755.2041	756.2113	Negative	C_33_H_40_O_20_	0.07549	0.26	0.34
Sophoraflavanone D	5.425	439.1754	440.1835	Negative	C_25_H_28_O_7_	1.75629	1.85	2.01
Quercetin 3-rhamnoside-3′-sulfate	5.431	527.0502	528.0574	Negative	C_21_H_20_O_14_S	0.20052	0.55	0.34
Quercetin 3-(4″-malonylrhamnoside)	5.435	533.0936	534.101	Negative	C_24_H_22_O_14_	0.20509	2.21	3.51
Quercetin 3-*O*-glucosyl-rhamnosyl-glucoside	5.471	771.1994	772.2062	Negative	C_33_H_40_O_21_	0.65686	0.69	0.59
Quercetin 3-*O*-(6″-galloyl)-beta-*D*-galactopyranoside	5.514	615.0992	616.1064	Negative	C_28_H_24_O_16_	0.02731	1.73	2.28
Quercetin 3-(2″-galoylrutinoside)	5.529	761.1573	762.1644	Negative	C_34_H_34_O_20_	0.32181	1.51	1.76
Naringin Dihydrochalcone	5.581	581.1873	582.1949	Negative	C_27_H_34_O_14_	0.43292	0.52	0.67
Quercetin 3-(2″-galloyl-alpha-*L*-arabinopyranoside)	5.619	585.0886	586.0959	Negative	C_27_H_22_O_15_	0.03799	2.06	3.48
Kaempferol-3-*O*-(6″-galloyl)-beta-glucopyranoside	5.713	599.1041	600.1115	Negative	C_28_H_24_O_15_	0.15739	1.69	1.86
Luteolin	5.789	285.0403	286.0477	Negative	C_15_H_10_O_6_	0.5884	0.36	0.59
Kaempferol 3-*O*-alpha-*L*-rhamnopyranosyl-(1→2)-alpha-*L*-rhamnopyranoside	5.799	577.1572	578.1636	Negative	C_27_H_30_O_14_	1.53638	0.619	0.58
Quercetin 3-(4″-acetylrhamnoside)	5.824	489.1036	490.1111	Negative	C_23_H_22_O_12_	0.38542	0.45	0.22
Eriodictyol	5.827	287.0563	288.0634	Negative	C_15_H_12_O_6_	0.79821	0.53	1.33
Quercetin 3-(3″,6″-diacetylgalactoside)	5.846	547.1102	548.1166	Negative	C_25_H_24_O_14_	1.67053	1.33	1.99
Kaempferol 3-(4″-acetylrhamnoside)	5.979	473.109	474.1162	Negative	C_23_H_22_O_11_	0.12011	0.35	0.18
Kaempferol 3-(3″,4″-diacetylglucoside)	6.033	531.115	532.1217	Negative	C_25_H_24_O_13_	1.20898	1.43	1.62
Naringenin	6.043	271.0611	272.0685	Negative	C_15_H_12_O_5_	0.39687	0.61	0.62
Quercetin 3-(3″-acetyl-alpha-*L*-arabinofuranoside)	6.289	475.0878	476.0955	Negative	C_22_H_20_O_12_	0.84261	0.70	0.55
Pinocembrin	6.542	255.066	256.0736	Negative	C_15_H_12_O_4_	1.071	0.44	0.47
3-*O*-Methylgalangin	6.847	283.0611	284.0685	Negative	C_16_H_12_O_5_	0.33945	0.69	0.65
Quercetin 3-(2″-galloylrhamnoside)	7.519	599.1042	600.1115	Negative	C_28_H_24_O_15_	0.0438	1.26	2.19
6-Hydroxyluteolin 7-xyloside	8.682	433.0776	434.0849	Negative	C_20_H_18_O_11_	0.03295	0.55	0.99
**Amino acids**								
Glu-Lys-His	1.187	413.2134	412.207	Positive	C_17_H_28_N_6_O_6_	2.36594	0.51	0.31
5-oxoproline	1.527	130.0499	129.0426	Positive	C_5_H_7_NO_3_	0.37372	0.67	0.59
Asparaginyl-Valine	2.776	230.1141	231.1219	Negative	C_9_H_17_N_3_O_4_	2.02406	0.59	0.59
*N*-Acetylproline	5.047	156.0667	157.0739	Negative	C_7_H_11_NO_3_	0.74434	0.25	0.28
4-Bis(2-hydroxyethyl)amino-*L*-phenylalanine	5.079	269.15	268.1423	Positive	C_13_H_20_N_2_O_4_	1.5965	0.47	0.46
Leu-Ala-Asp	5.216	316.1514	317.1587	Negative	C_13_H_23_N_3_O_6_	0.03337	0.46	0.48
Glu-Ile-Ser	5.34	346.1618	347.1693	Negative	C_14_H_25_N_3_O_7_	0.47465	0.64	0.72
*L*-Leucyl-L-Valine	5.337	229.1553	230.163	Negative	C_11_H_22_N_2_O_3_	1.92326	0.62	0.79
*N*-Fructosyl glutamylphenylalanine	5.427	457.1822	456.1744	Positive	C_20_H_28_N_2_O_10_	1.15892	0.65	0.48
*N*-Acetyl-leucyl-leucine	5.96	285.1821	286.1893	Negative	C_14_H_26_N_2_O_4_	0.62236	0.43	0.35
**Phenolic acids and their derivatives**								
Gallic acid	3.268	169.0141	170.0215	Negative	C_7_H_6_O_5_	0.77336	1.48	3.10
Theogallin	4.096	343.0665	344.0744	Negative	C_14_H_16_O_10_	1.6446	2.98	6.48
4-p-Coumaroylquinic acid	5.252	337.0927	338.1002	Negative	C_16_H_18_O_8_	0.42346	0.98	0.56
5-Feruloylquinic acid	5.289	367.1032	368.1107	Negative	C_17_H_20_O_9_	0.59097	0.64	0.58
2,3-Dihydroxy-4-methoxybenzoic acid	5.349	183.0298	184.0372	Negative	C_8_H_8_O_5_	0.35565	1.83	2.15
p-Coumaric acid glucuronide	5.444	339.0722	340.0794	Negative	C_15_H_16_O_9_	0.29741	0.56	0.50
4-Coumaroylshikimate	5.513	319.0823	320.0896	Negative	C_16_H_16_O_7_	0.04986	0.48	0.33
**Tannins**								
Ginnalin A	5.279	467.0829	468.0904	Negative	C_20_H_20_O_13_	0.44861	3.06	2.54
3-Methylellagic acid 8-(3-acetylrhamnoside)	5.297	503.0832	504.0904	Negative	C_23_H_20_O_13_	0.12626	0.61	1.03
Pentagalloylglucose	5.305	939.111	940.1182	Negative	C_41_H_32_O_26_	0.07094	2.37	3.38
2-*O*-cinnamoyl-1-*O*-galloyl-beta-*D*-glucose	5.798	461.109	462.1162	Negative	C_22_H_22_O_11_	0.20785	1.18	0.56
**Other compounds**								
L-Ascorbic acid	1.498	175.0247	176.0321	Negative	C_6_H_8_O_6_	0.47818	0.61	0.43
Pyrogallol-2-*O*-glucuronide	1.532	301.0567	302.0638	Negative	C_12_H_14_O_9_	0.74231	0.45	0.28
3′,4′-Dihydroxyacetophenone	5.449	151.0401	152.0474	Negative	C_8_H_8_O_3_	0.42432	0.53	1.04
(*E*)-Osmundacetone	5.603	177.0556	178.063	Negative	C_10_H_10_O_3_	0.41601	2.45	2.48
Ailanthone	5.82	375.1442	376.1522	Negative	C_20_H_24_O_7_	1.75919	0.51	1.44
Angelicain	5.849	273.0766	292.0947	Negative	C_15_H_16_O_6_	0.88305	0.19	0.34
*L*-Citronellol glucoside	5.904	317.1971	318.2042	Negative	C_16_H_30_O_6_	0.37809	3.69	2.32
Acetovanillone	5.968	225.0765	166.063	Negative	C_9_H_10_O_3_	2.22552	0.51	0.36
Ketobemidone	6.449	248.1649	247.1572	Positive	C_15_H_21_NO_2_	1.28816	0.59	0.57

Note: Fold changes were calculated as the ratio of mean relative abundances (e.g., GT/BT indicates the mean relative abundance of GT divided by that of BT). Bold text indicates the chemical classes of the detected compounds.

## Data Availability

The data presented in this study are available on request from the corresponding author.

## Supplementary Materials

The following supporting information can be downloaded at: https://www.mdpi.com/article/10.3390/metabo16100760/s1, Figure S1: TIC overlay chromatograms of QC samples in positive and negative ion modes. (A) Positive ion mode; (B) negative ion mode; Figure S2: Representative chromatogram of quercetin and kaempferol. (A) Chromatogram of quercetin (40 μg/mL); (B) Chromatogram of kaempferol (40 μg/mL); Figure S3: Effects of TLTs on food intake and water consumption in HFD-induced obese mice. Columns with different letters are significantly different (*p* < 0.05); Table S1: Linear equations and correlation coefficients (R^2^) of key metabolites; Table S2: Linear equations and correlation coefficients (R^2^) of main active components; Table S3: The composition of the experimental diets; Table S4: Detailed annotation information of differential metabolites among three *Toona sinensis* leaf teas; Table S5: Exploratory correlations of candidate components in TLTs with obesity-related indicators. Methods S1: UPLC operating parameters.
