# Supplementary material for: Cultivar-Dependent Metabolite Signatures and Effects on Lipid Metabolism of Teas Processed from Three Varieties of Toona sinensis
Source: Metabolites. 2026 Oct 9;16(10):760. doi: 10.3390/metabo16100760 (PMC13707263; doi:10.3390/metabo16100760)
Supplement: Supplementary file 1 [file metabolites-16-00760-s001.zip › metabolites-4494643-supplementary.pdf]

Supplementary Material

Methods S1 UPLC operating parameters

Chromatographic separation was performed on an ACQUITY UPLC HSS T3 column (150 mm × 2.1 mm, 1.8 μm, Thermo Scientific). The mobile phase consisted of eluent A (acetonitrile/water, 5:95, v/v) and eluent B (water containing 0.1% formic acid/acetonitrile, 5:95, v/v). The column temperature was maintained at 30°C, and the injection volume was 2 μL. The gradient program was carried out at a flow rate of 0.4 mL/min as follows: 0–2% B over 1 min, 2–15% B over 1 min, 15–26% B over 6 min, 26–100% B over 1 min, held at 100% B for 1 min, then returned to 2% B over 1 min, followed by a 1-min re-equilibration at 2% B. All calibration curves showed excellent linearity ($R^2 > 0.998$) across the tested concentration ranges for all reference standards (Table S1).

Table S1. Linear equations and correlation coefficients (R^2) of key metabolites

Compound	Linear equation	R^2
Quercetin	y = 27489.2x - 7417.2	0.999
Kaempferol	y = 23661.0x - 3687.6	0.998

Note: x, amount of each analyzed injection compound (μg/mL); y, peak area.

Table S2. Linear equations and correlation coefficients (R^2) of main active components

Compound	Linear equation	R^2
Total flavonoids	y = 0.0039x + 0.0676	0.992
Total polyphenols	y = 0.0052x + 0.0519	0.997
Free amino acids	y = 7.2743x + 0.0456	0.991
Soluble protein	y = 0.0047x + 0.0417	0.993

Note: x, amount of each analyzed injection compound (mg/mL); y, Abs.

Table S3. The composition of the experimental diets

**Ingredient (g)**	**low-fat diet**	**high-fat diet**
Casein	191	262
Cornstarch	484	0
Dextrin	120	164
Sucrose	66	90
Soybean oil	24	33
Lard	19	321
Cellulose	48	65
Mineral mix	33	46
Vitamin mix	10	13
L-Cystine	3	4
Line bitartrate	2	3
TBHQ	0.01	0.07
Total	1000	1000
Total energy		
Protein, %	20.6	19
Fat, %	12.0	60
Carbohydrate, %	67.4	21
Energy, Kcal/g	3.616	5.2

Table S4. Detailed annotation information of differential metabolites among three *Toona sinensis* leaf teas

Compound Name	RT (min)	m/z	Formula	MS^2 Fragments	MSI Confidence Level
**Flavonoids and flavonoid glycosides**					
Morusinol	1.422	437.1599	$C_{25}H_{26}O_7$	393, 349, 285, 269, 241	3
Luteolin 6-C-glucoside 8-C-arabinoside	1.807	609.1452	$C_{27}H_{30}O_{16}$	447, 429, 369, 357, 327, 285	2
Quercetin 3-(3''-sulfatoglucoside)	5.364	543.0451	$C_{21}H_{20}O_{15}S$	380，301，151	2
Quercetin 3-rhamnosyl-(1->2)-rhamnosyl-(1->6)-glucoside	5.422	755.2041	$C_{33}H_{40}O_{20}$	609, 463, 301	2
Sophoraflavanone D	5.425	439.1754	$C_{25}H_{28}O_7$	421, 395, 353, 203, 175, 165	2
Quercetin 3-rhamnoside-3'-sulfate	5.431	527.0502	$C_{21}H_{20}O_{14}S$	447, 301	2
Quercetin 3-(4''-malonylrhamnoside)	5.435	533.0936	$C_{24}H_{22}O_{14}$	491, 463, 300, 271	2
Quercetin 3-O-glucosyl-rhamnosyl-glucoside	5.471	771.1994	$C_{33}H_{40}O_{21}$	609, 463, 301	2
Quercetin 3-O-(6''-galloyl)-beta-D-galactopyranoside	5.514	615.0992	$C_{28}H_{24}O_{16}$	463, 301	1
Quercetin 3-(2''-galoylrutinoside)	5.529	761.1573	$C_{34}H_{34}O_{20}$	609, 463, 301	2
Naringin Dihydrochalcone	5.581	581.1873	$C_{27}H_{34}O_{14}$	437, 273, 167, 123	1
Quercetin 3-(2''-galloyl-alpha-L-arabinopyranoside)	5.619	585.0886	$C_{27}H_{22}O_{15}$	433, 301	2
Kaempferol-3-O-(6''-galloyl)-beta-glucopyranoside	5.713	599.1041	$C_{28}H_{24}O_{15}$	447, 285	1
Luteolin	5.789	285.0403	$C_{15}H_{10}O_6$	199, 175, 151, 133	1
Kaempferol 3-O-alpha-L-rhamnopyranosyl-(1->2)-alpha-L-rhamnopyranoside	5.799	577.1572	$C_{27}H_{30}O_{14}$	431, 285	2
Quercetin 3-(4''-acetylrhamnoside)	5.824	489.1036	$C_{23}H_{22}O_{12}$	447, 301	2
Eriodictyol	5.827	287.0563	$C_{15}H_{12}O_6$	271, 151, 135	1
Quercetin 3-(3'',6''-diacetylgalactoside)	5.846	547.1102	$C_{25}H_{24}O_{14}$	505, 463, 301	2
Kaempferol 3-(4''-acetylrhamnoside)	5.979	473.109	$C_{23}H_{22}O_{11}$	431, 285	2
Kaempferol 3-(3'',4''-diacetylglucoside)	6.033	531.115	$C_{25}H_{24}O_{13}$	489, 447, 285	2
Naringenin	6.043	271.0611	$C_{15}H_{12}O_5$	151, 119, 107	1
Quercetin 3-(3''-acetyl-alpha-L-arabinofuranoside)	6.289	475.0878	$C_{22}H_{20}O_{12}$	433, 301	2
Pinocembrin	6.542	255.066	$C_{15}H_{12}O_4$	213, 151	1
3-O-Methylgalangin	6.847	283.0611	$C_{16}H_{12}O_5$	268, 239, 211, 183, 168	1
Quercetin 3-(2''-galloylrhamnoside)	7.519	599.1042	$C_{28}H_{24}O_{15}$	447, 301	2
6-Hydroxyluteolin 7-xyloside	8.682	433.0776	$C_{20}H_{18}O_{11}$	301	2
**Amino acids**					3
Glu-Lys-His	1.187	413.2134	$C_{17}H_{28}N_6O_6$	284, 258, 156. 84	1

5-oxoproline	1.527	130.0499	$C_5H_7NO_3$	130, 84	2
Asparaginyl-Valine	2.776	230.1141	$C_9H_{17}N_3O_4$	230, 146, 115, 72	1
N-Acetylproline	5.047	156.0667	$C_7H_{11}NO_3$	112, 70	1
4-Bis(2-hydroxyethyl)amino-L-phenylalanine	5.079	269.15	$C_{13}H_{20}N_2O_4$	221, 191, 146, 120	3
Leu-Ala-Asp	5.216	316.1514	$C_{13}H_{23}N_3O_6$	205, 134, 86	2
Glu-Ile-Ser	5.34	346.1618	$C_{14}H_{25}N_3O_7$	243, 219, 130, 106	2
L-Leucyl-L-Valine	5.337	229.1553	$C_{11}H_{22}N_2O_3$	185, 130, 86	1
N-Fructosyl glutamylphenylalanine	5.427	457.1822	$C_{20}H_{28}N_2O_{10}$	323, 294, 179	2
N-Acetyl-leucyl-leucine	5.96	285.1821	$C_{14}H_{26}N_2O_4$	226, 185, 143, 86	2
**Phenolic acids and their derivatives**					
Gallic acid	3.268	169.0141	$C_7H_6O_5$	125, 97, 79	1
Theogallin	4.096	343.0665	$C_{14}H_{16}O_{10}$	191, 169, 125	1
4-p-Coumaroylquinic acid	5.252	337.0927	$C_{16}H_{18}O_8$	191, 173, 163, 119	1
5-Feruloylquinic acid	5.289	367.1032	$C_{17}H_{20}O_9$	193, 191, 173, 134	1
2,3-Dihydroxy-4-methoxybenzoic acid	5.349	183.0298	$C_8H_8O_5$	140, 125, 96	1
p-Coumaric acid glucuronide	5.444	339.0722	$C_{15}H_{16}O_9$	163, 145, 119	2
4-Coumaroylshikimate	5.513	319.0823	$C_{16}H_{16}O_7$	163, 119	2
**Tannins**					
Ginnalin A	5.279	467.0829	$C_{20}H_{20}O_{13}$	315, 169, 125	2
3-Methylellagic acid 8-(3-acetylrhamnoside)	5.297	503.0832	$C_{23}H_{20}O_{13}$	461, 315, 301	3
Pentagalloylglucose	5.305	939.111	$C_{41}H_{32}O_{26}$	787, 769, 617, 599, 465, 313, 169	1
2-O-cinnamoyl-1-O-galloyl-beta-D-glucose	5.798	461.109	$C_{22}H_{22}O_{11}$	313, 169, 131	1
**Other compounds**					
L-Ascorbic acid	1.498	175.0247	$C_6H_8O_6$	115, 87, 71	1
Pyrogallol-2-O-glucuronide	1.532	301.0567	$C_{12}H_{14}O_9$	125, 113	3
3',4'-Dihydroxyacetophenone	5.449	151.0401	$C_8H_8O_3$	123, 108, 95	1
(E)-Osmundacetone	5.603	177.0556	$C_{10}H_{10}O_3$	149, 135, 121	1
Ailanthone	5.82	375.1442	$C_{20}H_{24}O_7$	357, 339, 329, 311, 299	3
Angelicain	5.849	273.0766	$C_{15}H_{16}O_6$	273, 249, 231, 217	1
L-Citronellol glucoside	5.904	317.1971	$C_{16}H_{30}O_6$	155, 137, 119, 101	2
Acetovanillone	5.968	225.0765	$C_9H_{10}O_3$	137, 122, 109, 94	1
Ketobemidone	6.449	248.1649	$C_{15}H_{21}NO_2$	228, 214, 200, 186, 158	2

Table S5. Exploratory correlations of candidate components in TLTs with obesity-related indicators

**Compound Name**	Body weight	Liver weight	IngWAT weight	EpiWAT weight	Adipocyte area of IngWAT	Adipocyte area of EpiWAT	T-CHO	LDL-C	TG
Quercetin 3-(3''-sulfatoglucoside)	-0.342	-0.470	-0.435	-0.554	-0.705	-0.597	-0.184	-0.636	-0.815
Quercetin 3-rhamnosyl-(1->2)-rhamnosyl-(1->6)-glucoside	-0.372	-0.514	-0.453	-0.619	-0.735	-0.601	-0.310	-0.667	-0.799
Naringin Dihydrochalcone	-0.320	-0.463	-0.496	-0.624	-0.816	-0.624	-0.305	-0.690	-0.765
Luteolin	-0.496	-0.637	-0.673	-0.637	-0.939	-0.652	-0.338	-0.843	-0.832
Naringenin	-0.434	-0.523	-0.453	-0.444	-0.752	-0.688	-0.096	-0.582	-0.869
Pinocembrin	-0.418	-0.523	-0.505	-0.499	-0.758	-0.633	-0.163	-0.625	-0.858
N-Acetylproline	-0.459	-0.516	-0.523	-0.532	-0.752	-0.668	-0.197	-0.627	-0.866
L-Leucyl-L-Valine	-0.455	-0.501	-0.594	-0.661	-0.864	-0.689	-0.335	-0.747	-0.816
4-Coumaroylshikimate	-0.403	-0.489	-0.447	-0.525	-0.747	-0.640	-0.213	-0.599	-0.834
ginnalin A	0.367	0.627	0.605	0.553	0.861	0.578	0.249	0.794	0.889
Angelicain	-0.484	-0.470	-0.582	-0.574	-0.802	-0.591	-0.253	-0.701	-0.872
L-Citronellol glucoside	0.577	0.518*	0.799	0.694	0.903	0.681	0.332	0.778	0.884
Quercetin	-0.377	-0.462	-0.766	-0.511	-0.738	-0.466	-0.236	-0.755	-0.638
Kaempferol	-0.479	-0.545	-0.801	-0.643	-0.899	-0.570	-0.353	-0.846	-0.773
Total flavonoids	-0.476	-0.504	-0.768	-0.598	-0.867	-0.586	-0.292	-0.796	-0.735
Total polyphenols	-0.524	-0.601	-0.792	-0.678	-0.936	-0.645	-0.386	-0.868	-0.856
Free amino acids	-0.560	-0.564	-0.650	-0.631	-0.841	-0.650	-0.319	-0.746	-0.849
Soluble protein	-0.195	-0.325	-0.551	-0.482	-0.605	-0.530	-0.035	-0.611	-0.814

Note: Correlation coefficients (r) were calculated based on cultivar-level mean values (n = 3 per cultivar, total 9 data points). This table is for descriptive visualization of co-variation trends only; no formal statistical inference should be drawn. The candidate metabolites identified here require further validation.

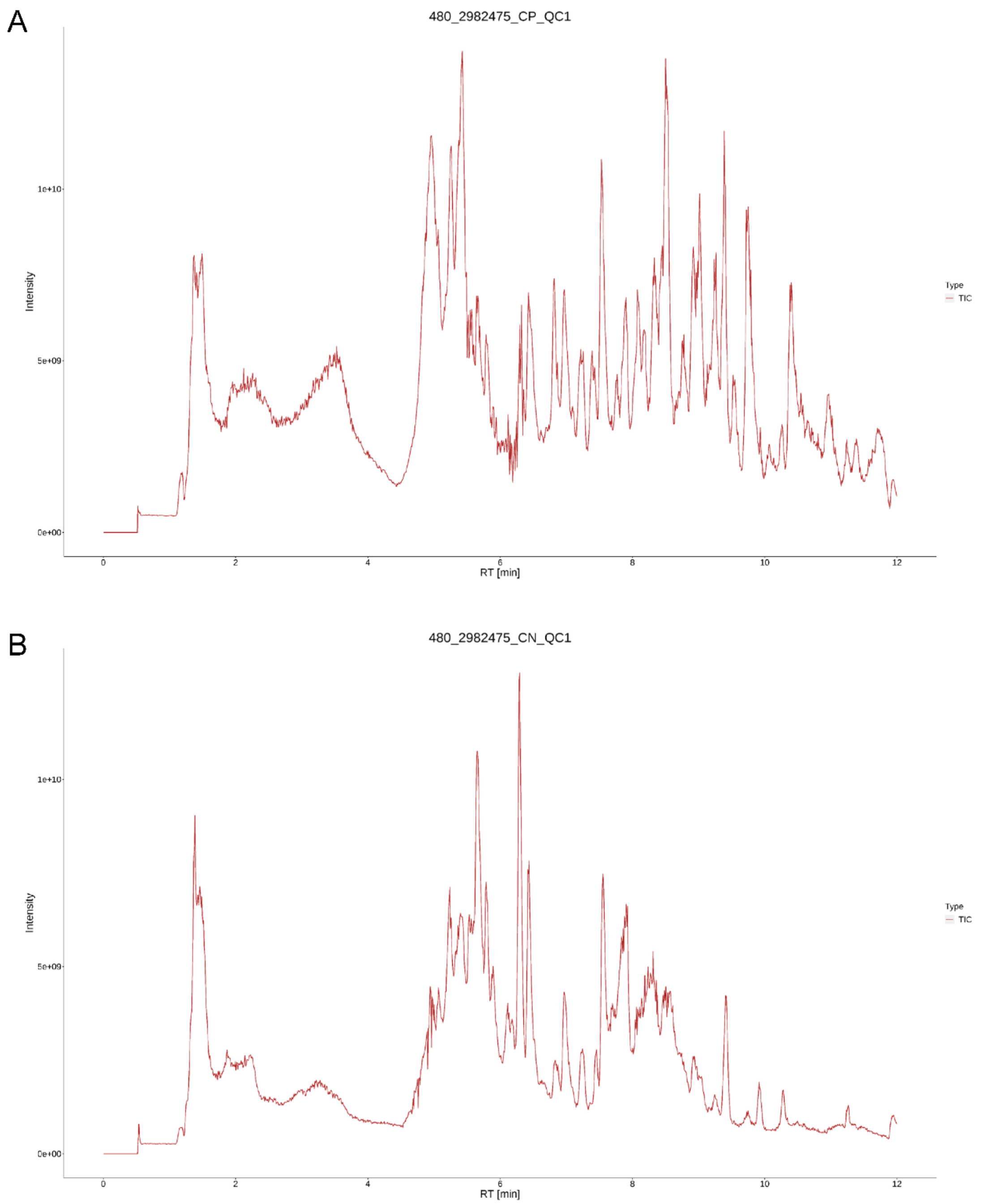

Figure S1. TIC overlay chromatograms of QC samples in positive and negative ion modes. (A) Positive ion mode; (B) negative ion mode.

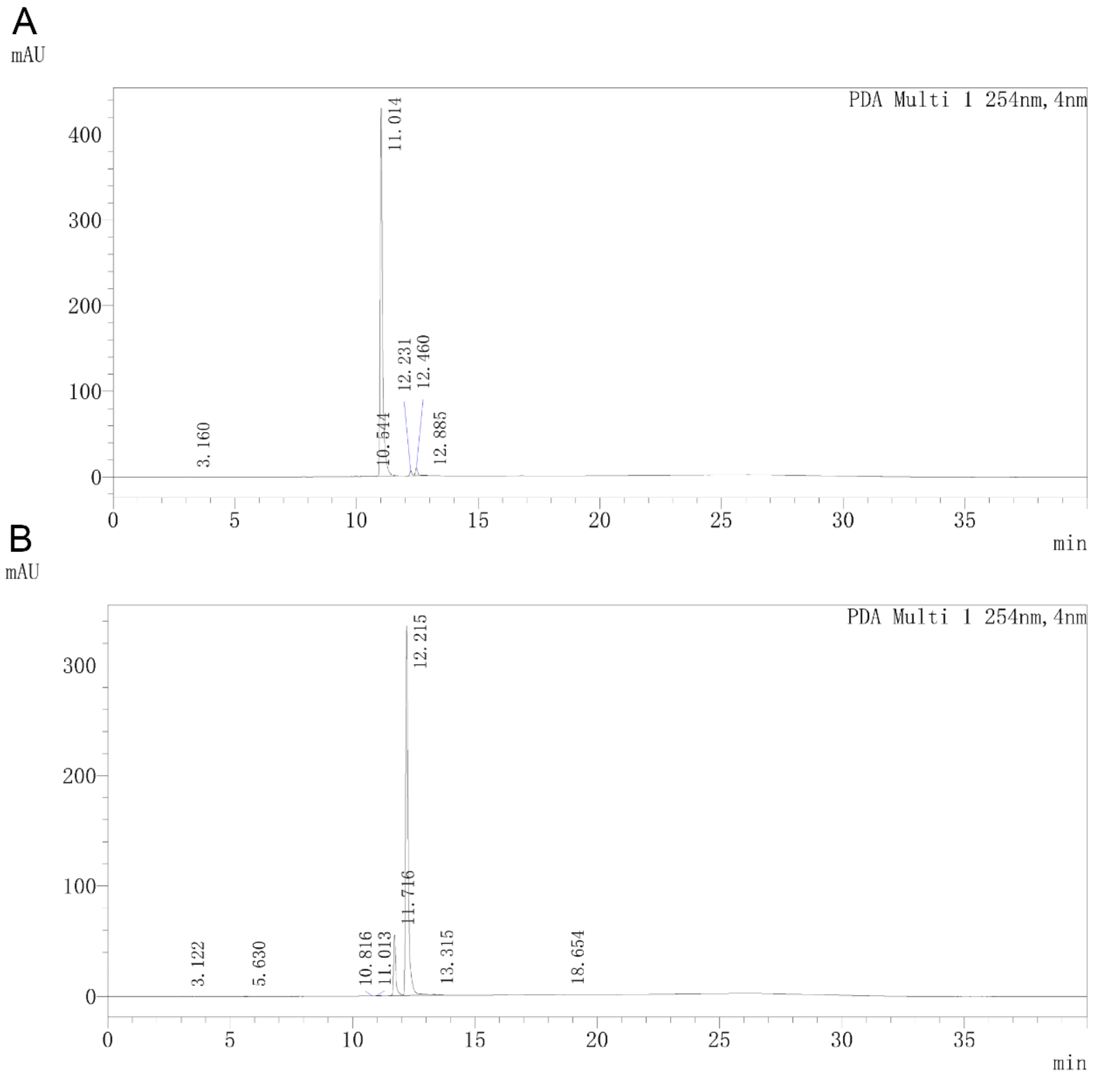

Figure S2. Representative chromatogram of quercetin and kaempferol. (A) Chromatogram of quercetin (40 µg/mL); (B) Chromatogram of kaempferol (40 µg/mL).

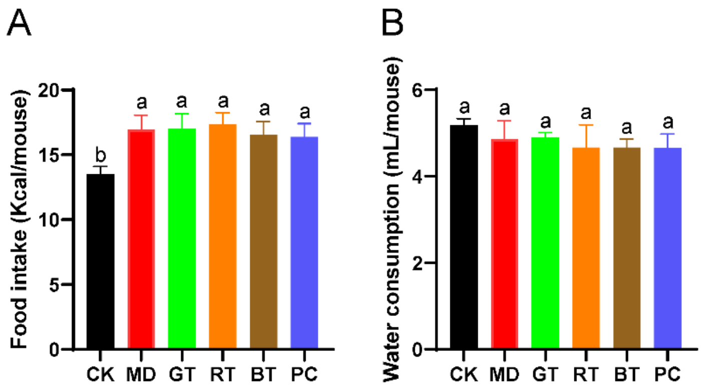

Figure S3. Effects of TLTs on food intake and water consumption in HFD-induced obese mice. Columns with different letters are significantly different ($p < 0.05$).
